# Systematic sequencing of mRNA from the Antarctic krill (*Euphausia superba*) and first tissue specific transcriptional signature

**DOI:** 10.1186/1471-2164-9-45

**Published:** 2008-01-28

**Authors:** Cristiano De Pittà, Cristiano Bertolucci, Gabriella M Mazzotta, Filippo Bernante, Giorgia Rizzo, Barbara De Nardi, Alberto Pallavicini, Gerolamo Lanfranchi, Rodolfo Costa

**Affiliations:** 1 CRIBI Biotechnology Centre, Università degli Studi di Padova, Via U. Bassi, 58/B, 35121, Padova, Italy; 2 Dipartimento di Biologia, Università degli Studi di Padova, Via U. Bassi, 58/B, 35121, Padova, Italy; 3 Dipartimento di Biologia ed Evoluzione, Università degli Studi di Ferrara, Via L. Borsari, 46, 44100 Ferrara, Italy; 4 Dipartimento di Biologia, Università degli Studi di Trieste, P.le Valmaura, 9, 34148 Trieste, Italy

## Abstract

**Background:**

Little is known about the genome sequences of Euphausiacea (krill) although these crustaceans are abundant components of the pelagic ecosystems in all oceans and used for aquaculture and pharmaceutical industry. This study reports the results of an expressed sequence tag (EST) sequencing project from different tissues of *Euphausia superba *(the Antarctic krill).

**Results:**

We have constructed and sequenced five cDNA libraries from different Antarctic krill tissues: head, abdomen, thoracopods and photophores. We have identified 1.770 high-quality ESTs which were assembled into 216 overlapping clusters and 801 singletons resulting in a total of 1.017 non-redundant sequences. Quantitative RT-PCR analysis was performed to quantify and validate the expression levels of ten genes presenting different EST countings in krill tissues. In addition, bioinformatic screening of the non-redundant *E. superba *sequences identified 69 microsatellite containing ESTs. Clusters, consensuses and related similarity and gene ontology searches were organized in a dedicated *E. superba *database .

**Conclusion:**

We defined the first tissue transcriptional signatures of *E. superba *based on functional categorization among the examined tissues. The analyses of annotated transcripts showed a higher similarity with genes from insects with respect to Malacostraca possibly as an effect of the limited number of Malacostraca sequences in the public databases. Our catalogue provides for the first time a genomic tool to investigate the biology of the Antarctic krill.

## Background

Euphausiacea (krill) are small shrimplike crustaceans that are abundant in the pelagic ecosystems of all oceans. There are about 85 species of Euphausiacea, making this one of the smallest orders in the class of Malacostraca [1]. Phylogenetic analysis of the Eumalacostraca orders based on 28S rDNA sequences suggests that Euphausiacea are more closely related to Mysida than to the Decapoda [2].

In the Southern Ocean, krill is a critical link between primary productivity and most of the predators at higher trophic levels such as birds, fish, seals, squid and whales [3]. The krill biomass in the Southern Ocean has been estimated at 400–1550 million tons with sustainable annual harvest at around 70–200 million tons. Therefore, krill biomass that could be available for human food is comparable to the biomass of all the other aquatic species currently fished by humans, but only six species of krill are at present harvested commercially [4,5]. Commercial fishing of krill is done in the Southern Ocean and around Japan. The global annual production amounts to 150 – 200.000 tons, most of this from the Scotia Sea [6,7]. Most of the fished krill is used for aquaculture and aquarium feedings, as baits in sport fishing, or in the pharmaceutical industry.

The Antarctic krill (*Euphausia superba*, Dana 1852) has a circumpolar distribution with the highest concentrations in the Atlantic sector of the Southern Ocean. It is a key species of the Antarctic ecosystem and plays an important role both as feeder of algae, bacteria and micro-zooplancton and as a prey of vertebrates [8]. *E. superba *displays a large daily vertical migration that occurs generally within the upper 200 m water column making a significant amount of biomass available as food for predators near the surface at night and in deeper waters during the day [9]. Basic knowledge of crustacean biology is limited by the lack of information about their genomes. Considering all orders in the class of Malacostraca, no genome has yet been fully sequenced. At present Genbank carries 116,640 nucleotide and 11,932 protein sequences (Table 1), with a high rate of redundancy. Currently only 434 nucleotide and 310 protein sequences have been identified in Euphausiacea (GenBank source, release of November 2007). Specifically for *E. superba *only 69 nucleotide and 17 amino acid sequences have been obtained; they identify key proteins and enzymes of oxidative phosphorylation (*NADH dehydrogenase subunit 1*, *2*, *3*, *4*, *4L*, *5*; *Cytochrome oxidase subunit I*, *II*, *III*; *ATP synthase subunit 6*; *cytochrome b*; *cytochrome b apoenzyme *and *cytochrome c oxidase subunit I*) and of phototransduction (*opsin*). In the subphylum Crustacea there are 33 complete (or nearly complete) mitochondrial DNA sequences: 4 Branchiopoda, 8 Maxillopoda, 18 Malacostraca, and one of Ostracoda, Cephalocarida and Remipedia (Table 2). In a previous investigation Machida *et al*. [10] determined the nearly complete DNA sequence of the mitochondrial genome of *E. superba *(14,606 bp).

**Table 1:** Nucleotide and protein sequences belonging to all orders of the Malacostraca class available from public databases at November 2007.

**Class**	**Subclass**	**Superorder**	**Order or Sub-order**	**Nucleotide sequences**	**Protein sequences**
Malacostraca	Eumalacostraca	Eucarida	Decapoda	97,083	7,862
Malacostraca	Eumalacostraca	Eucarida	Euphausiacea	434	310
Malacostraca	Eumalacostraca	Hoplocarida	Stomatopoda	119	214
Malacostraca	Eumalacostraca	Peracarida	Amphipoda	14,732	1,745
Malacostraca	Eumalacostraca	Peracarida	Cumacea	93	82
Malacostraca	Eumalacostraca	Peracarida	Isopoda	3,355	1,155
Malacostraca	Eumalacostraca	Peracarida	Mictacea	1	0
Malacostraca	Eumalacostraca	Peracarida	Mysidacea	771	561
Malacostraca	Eumalacostraca	Peracarida	Spelaeogriphacea	1	0
Malacostraca	Eumalacostraca	Peracarida	Tanaidacea	18	1
Malacostraca	Eumalacostraca	Peracarida	Thermosbaenacea	4	0
Malacostraca	Eumalacostraca	Syncarida	Anaspidacea	27	2
Malacostraca	Eumalacostraca	Syncarida	Bathynellacea	2	0

**Total**				**116,640**	**11,932**

**Table 2:** List of complete or nearly complete mitochondrial DNA sequences of the subphylum Crustacea available from public databases at November 2007.

**#**	**Class**	**Subclass**	**Organism**	**Refseq**	**Reference**
1	Branchiopoda	Phyllopoda	*Triops longicaudatus*	NC_006079	[50]
2	Branchiopoda	Sarsostraca	*Artemia franciscana*	NC_001620	Unpublished
3	Branchiopoda	Phyllopoda	*Daphnia pulex*	NC_000844	[51]
4	Branchiopoda	Phyllopoda	*Triops cancriformis*	NC_004465	[52]
5	Cephalocarida	Brachypoda	*Hutchinsoniella macracantha*	NC_005937	[53]
6	Malacostraca	Eumalacostraca	*Halocaridina rubra*	NC_008413	[54]
7	Malacostraca	Eumalacostraca	*Geothelphusa dehaani*	NC_007379	[55]
8	Malacostraca	Eumalacostraca	*Ligia oceanica*	NC_008412	[56]
9	Malacostraca	Eumalacostraca	*Squilla mantis*	NC_006081	[50]
10	Malacostraca	Eumalacostraca	*Harpiosquilla harpax*	NC_006916	[57]
11	Malacostraca	Eumalacostraca	*Pseudocarcinus gigas*	NC_006891	[58]
12	Malacostraca	Eumalacostraca	*Macrobrachium rosenbergii*	NC_006880	[58]
13	Malacostraca	Eumalacostraca	*Callinectes sapidus*	NC_006281	Unpublished
14	Malacostraca	Eumalacostraca	*Squilla empusa*	NC_007444	Unpublished
15	Malacostraca	Eumalacostraca	*Lysiosquillina maculata*	NC_007443	Unpublished
16	Malacostraca	Eumalacostraca	*Gonodactylus chiragra*	NC_007442	Unpublished
17	Malacostraca	Eumalacostraca	*Marsupenaeus japonicus*	NC_007010	[59]
18	Malacostraca	Eumalacostraca	*Eriocheir sinensis*	NC_006992	[60]
19	Malacostraca	Eumalacostraca	*Cherax destructor*	NC_011243	[61]
20	Malacostraca	Eumalacostraca	*Panulirus japonicus*	NC_004251	[62]
21	Malacostraca	Eumalacostraca	*Portunus trituberculatus*	NC_005037	[63]
22	Malacostraca	Eumalacostraca	*Pagurus longicarpus*	NC_003058	[64]
23	Malacostraca	Eumalacostraca	*Penaeus monodon*	NC_002184	Unpublished
24	Maxillopoda	Copepoda	*Tigriopus californicus*	NC_008831	Unpublished
25	Maxillopoda	Cirripedia	*Pollicipes mitella*	NC_008742	Unpublished
26	Maxillopoda	Branchiura	*Argulus americanus*	NC_005935	[50]
27	Maxillopoda	Copepoda	*Tigriopus japonicus*	NC_003979	[65]
28	Maxillopoda	Copepoda	*Lepeophtheirus salmonis*	NC_007215	[66]
29	Maxillopoda	Cirripedia	*Megabalanus volcano*	NC_006293	Unpublished
30	Maxillopoda	Cirripedia	*Pollicipes polymerus*	NC_005936	[53]
31	Maxillopoda	Cirripedia	*Tetraclita japonica*	NC_008974	Unpublished
32	Ostracoda	Myodocopa	*Vargula hilgendorfii*	NC_005306	[67]
33	Remipedia	Nectiopoda	*Speleonectes tulumensis*	NC_005938	[53]

The identification of novel shrimp genes by systematic sequencing of genomic DNA is hindered by the dispersion of the genes among large non-coding regions and by the presence of introns within genes. Current genomics technologies, like SAGE [11], differential display [12] and systematic sequencing of expressed sequence tags [13,14], are very useful approaches to identify protein coding genes rapidly on a large scale. Moreover, the frequency of a given sequence in the SAGE or cDNA libraries can be related to the relative abundance of the corresponding mRNA, giving an indication of the level of gene expression [15].

The aim of our study was to significantly increase the number of krill genes in the public database and to discover tissue specific genes. For this purpose we have produced and sequenced five cDNA libraries from different Antarctic krill tissues: head, abdomen, thoracopods and photophores. We have developed special cDNA libraries optimized to directionally cloning full-length cDNA in plasmid vectors without enzymatic digestion. We have identified 1,770 high-quality EST clones that have been grouped in 1,017 different clusters. Of these, 309 clusters were successfully annotated while 708 did not show a significant similarity with known genes from other organisms. Clusters, consensus and related similarity and gene ontology searches were organized in a dedicated *E. superba *database [16].

## Results and Discussion

### Construction of cDNA libraries and EST analysis

**Figure 1:**
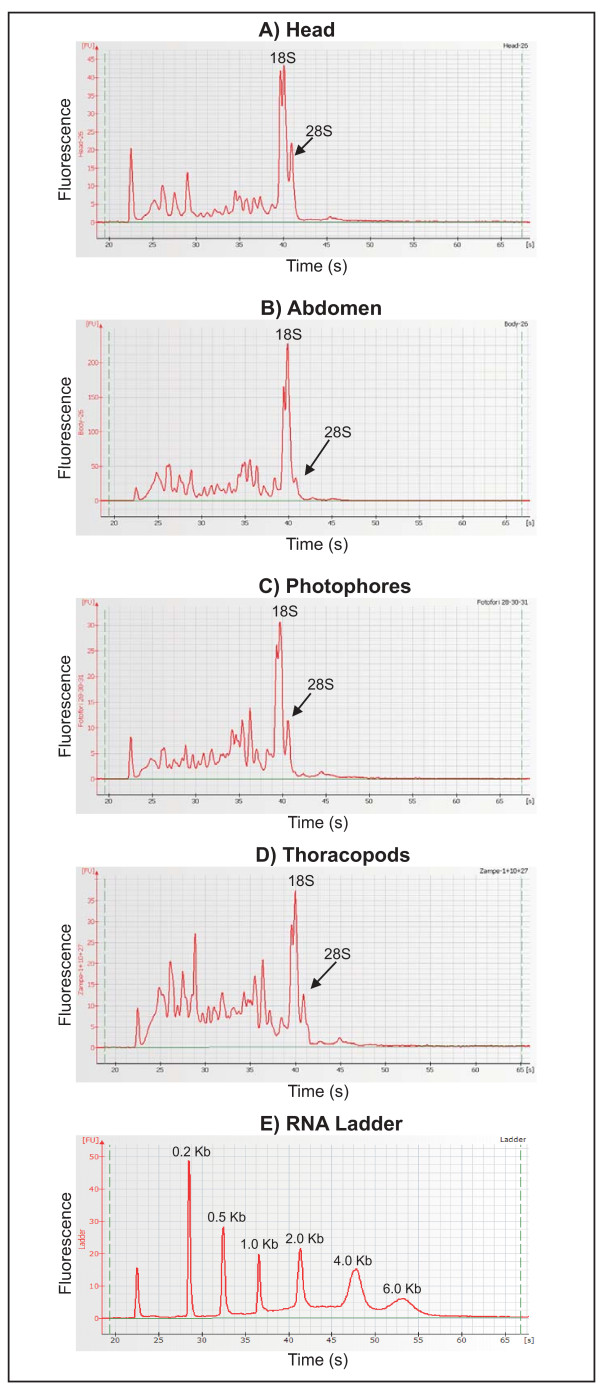
**Electropherograms of *E. superba *tissue-specific total RNAs**. (**A-D**) Electropherograms resulting from Agilent 2100 bioanalyzer analysis on total RNA extracted from head, abdomen, photophores and thoracopods. X-axis: time of ribosomal RNA peak appearance, corresponding to the size of the fragment; Y-axis: fluorescence of the peak, corresponding to its concentration. The size and the concentration of the sample peaks are calculated by the software via comparison with a RNA ladder at known concentration (**E**). *E. superba *RNA samples showed some products with a migration time between 22 and 35 seconds (from 200 bp to 1.000 bp) indicating a partial RNA degradation.

Total RNA was independently isolated from tissues (head, abdomen, thoracopods and photophores) dissected from specimens of *E. superba *collected at five different time points during 24 hours. The analysis of total RNA samples, performed by capillary electrophoresis, showed absence of genomic DNA contamination and a peculiar electropherogram as shown in Fig. 1. In particular, total RNAs show low molecular weight from 200 bp to 1 kb, perhaps as result of a partial RNA degradation.

Five independent tissue-specific cDNA libraries, named K01 and K05 (head), K06 (abdomen), K07 (photophores) and K09 (thoracopods), were produced from total RNA pools. For head we have sequenced only K05 cDNA library because it presented more recombinant clones compared to K01. Recombinant bacterial clones from each library were randomly picked and the EST were sequenced from the 5'-end. The average insert size for all libraries was estimated to be 412 bp.

### EST assembly and construction of an Antarctic krill transcript catalogue

A total of 2,046 ESTs were initially analyzed for sequence quality and vector sequences were recognized and deleted. Two-hundred-seventy-six low quality ESTs were removed and 1,770 (86.5%) high-quality ESTs were further processed. These ESTs assembled by similarity into 216 clusters and 801 singletons, resulting in a total of 1,017 non-redundant (consensus) sequences. A list of the sequencing trend for each cDNA library is presented in Table 3. Interestingly, we obtained a low percentage of clusters composed by ≥ 2 ESTs from cDNA libraries prepared from the krill head (K01, K05) showing that no specific transcripts are particularly enriched in this tissue. As expected from the above data, the head reveals a higher percentage (about 65%) of new putative transcripts, with respect to abdomen (52.3%), photophores (50.2%) and thoracopods (44.4%) in which the presence of enriched transcripts was instead revealed by EST sequencing.

**Table 3:** Results of EST assembly for each krill cDNA library.

**Library**	**Tissue**	**Total ESTs**	**Discarded ESTs**	**Analyzed ESTs**	**# GenBank**	**# EST in cluster**	**# cluster**	**# singletons**	**# consensus**	**% redundancy**	**% discovery**
**K01**	Head	35	9	26	ES542703–ES542728	12	10	14	24	38.46	61.54
**K05**	Head	579	124	455	ES542729–ES543183	225	108	230	338	35.16	64.84
**K06**	Abdomen	508	47	461	ES543184–ES543644	250	110	211	321	42.73	57.27
**K07**	Photophores	506	52	454	ES543645–ES544098	255	114	199	313	49.78	50.22
**K09**	Thoracopods	418	44	374	ES544099–ES544472	227	108	147	255	55.61	44.39
**Overall**		2046	276	1770		969	450	801	1251	44.35	55.65

Total ESTs = number of produced chromatograms; Discarded ESTs = number of low quality ESTs; Analyzed ESTs = number of sequences processed for clustering; # GenBank = accession numbers of ESTs deposited in GenBank; # EST in cluster = number of sequences in cluster; # clusters = total number of clusters; # singletons = number of putative transcripts identified by one EST; # consensus = number of non-redundant sequences; % redundancy = percentage of sequences not identified in an exclusive manner; % discovery = percentage of ESTs identifying putative new transcripts in the total EST analyzed.

The number of ESTs in each cluster varies from 2 (93 clusters) to 88 (1 cluster). The average length of a cluster is 436 bp with the longest assembled sequence being 1,417 bp and the shortest 153 bp. The 91% of cluster consensus contains the 3'-end region of mRNAs as demonstrated by the presence of a poly-adenylation signal.

Each non redundant sequence was searched in the nucleotides database and UniProtKB database using Blast-N and Blast-X with an *e*-value cut off of < e^-40 ^and < e^-10^, respectively. These values were empirically chosen considering the low amount of sequences data available for *Euphausiacea *and similar shrimp species and the need of stringency in providing a reliable catalogue of Antarctic krill genes. All annotations were further manually examined, in order to assign the best describing text to the correspondent cluster.

Overall, 70% of non-redundant sequences (708 out of 1,017), identified by about 50% of total produced ESTs, showed no or poor similarity matches, and they probably represent completely unknown Antarctic krill transcripts that could be characterized in future studies. Additional file 1 lists the 309 (30%) non-redundant sequences identifying known *Euphausia *genes or sequences showing significant similarity to genes from arthropods (63.4%) and other species (36.6%), such as *Homarus americanus *(3.2%), *Aedes aegypti *(12.0%), *Drosophila melanogaster *(3.9%), *Bombyx mori *(3.6%), *Brachydanio rerio *(4.9%), *Mus musculus *(3.9%), *Rattus norvegicus *(3.9%),* Homo sapiens *(4.5%). Antarctic krill sequences generally show a greater similarity to genes of insects (about 36%) than to genes of Malacostraca (about 23%) and only 7% were similar to the known sequences of Euphausiacea. This result could be due to the limited number of Malacostraca gene and protein sequences available in the public databases (*gene sequences: 116,666 and protein sequences: 11,941 at November 2007*) respect to insects (*gene sequences: 1,869,511 and protein sequences: 336,246 at November 2007*). The total collection of 1,770 *E. superba *3'-EST has been deposited in the EBI-GenBank-DBJ database (GenBank accession numbers from ES542703 to ES544472).

One of the most noticeable features of our EST catalogue is that mitochondrial transcripts are quite abundant (about 10% of the total ESTs): 84 ESTs (about 4.7%) matched with nine different *E. superba *mitochondrial transcripts (*ATPase6*,* COI*, *COII*, *COIII*, *cyt b*, *ND1*, *ND2*, *ND3*, *ND6*) and about 5% ESTs (88 out of 1,770) identified the large mitochondrial 16S rRNA gene. In future experiments, to avoid the repetitive sequencing of this abundant mRNA, we plan to introduce, during cDNA insert amplification, interference primers specifically designed for the 16S rRNA [17]. Moreover, we found a small percentage of ESTs (1.6%), showing similarity with 18S and 28S rRNA. About 2% of ribosomal RNAs contamination is common to other systematic sequencing projects.

The *E. superba *mitochondrial gene sequences are very similar to those commonly found in the mitochondrial genomes of other arthropods, including 13 protein-coding genes, 19 tRNA and 2 rRNA genes [10]. Machida *et al*. [10] have demonstrated that mitochondrial protein-coding genes are transcribed from the same DNA strand, left to right, except for *ND1*, *ND4L*, *ND4 *and *ND5 *genes, while two ribosomal RNA genes are encoded by L strands.

We did not find highly represented tissue-specific mRNAs, while some ribosomal proteins like L22 (31 ESTs), S25 (31), P1 (16), L37A (15), L24 (13), P2 (13), S14 (9), S14 (10), L34 (9) were expressed at the same level in all analyzed krill tissues. The only genuine tissue-specific mRNA appears to be *myosin light chain *(8 ESTs, cluster KRC00032) that is highly represented in a strictly committed tissue such as skeletal muscle localized in abdomen (library K06). Moreover, we found 3 unknown transcripts among the 20 most expressed genes: KRC00118 (10 ESTs), KRC00407 (9 ESTs) and KRC00101 (7 ESTs) that could be interesting for future functional studies.

Since very few abundant transcripts were found that could hamper the identification of rare transcripts, it seems plausible that random sequencing of our Antarctic krill libraries would continue to represent an effective strategy for identifying novel *E. superba *mRNAs.

### Functional categorization of *E. superba *ESTs

In order to facilitate functional genomic studies in Antarctic krill, 309 consensus sequences showing similarity with known genes or proteins were grouped into 14 functional categories (Table 4) according to Gene Ontology [18] and other resources developed for gene functional annotation [19]. A list of all annotated transcripts is shown in Additional file 1. A large majority of ESTs (20.51%), displaying putative identity with ribosomal sequences and genes for the translation machinery, were grouped in the translation functional category, characterized by 8% of all known transcripts. We found genes with regulative functions in the translational initiation, like *translation factor SUI1*,*initiation factor 4A and 3*, in the translational elongation like *elongation factor 1α*, *1β*, *2 *and a specific *tail muscle elongation factor 1γ*. Other abundant *E. superba *sequences fall into gene categories related to cell structure, cell motility and functional homeostasis. For instance, genes involved in the mechanisms of DNA transcription (1.47%), transport (1.97%), skeletal muscle contraction (3.4%), and in amino acid, fatty acid and carbohydrate metabolism (4.03 %) are comprised in this category. This class includes also SEC61 β-subunit, an important transport protein that plays a crucial role in the insertion of secretory and membrane polypeptides into the endoplasmic reticulum and cellular retinoic acid/retinol binding protein (RBP1), involved in the transport of retinol from the digestive gland to peripheral tissues [20]. A transcript included in this class (ID: KRC00589) shows a good similarity with hemocyanin, the main oxygen carrier molecule in arthropods and molluscs [21].

**Table 4:** Classification of the 1,017 krill unique consensus sequences in functional categories.

**#**	**Functional categories**	**Biological process**	**# Clusters**	**% Cluster**	**# ESTs**	**% ESTs**
**1**	**DNA replication & binding**	DNA replication	4	**0.79**	**8**	**0.45**
		DNA binding	4			
**2**	**Transcription**	Transcription process	2	**1.47**	**16**	**0.90**
		Regulation of transcription	8			
		Chromatine assembly and remodelling	5			
**3**	**Translation**	Translation	70	**8.06**	**363**	**20.51**
		Translation initiation	4			
		Translation elongation	7			
		Translation termination	1			
**4**	**Transport**	Electron transport	8	**1.97**	**32**	**1.81**
		Calcium ion transport	2			
		Intracellular protein transport	4			
		Transport	6			
**5**	**Metabolic processes**	Amino acid metabolic process	2	**4.03**	**73**	**4.12**
		Carbohydrate metabolic process	13			
		Fatty acid biosynthetic process	3			
		Various metabolic process	23			
**6**	**Proteolysis, protein folding and modification**	Proteolysis	16	**3.24**	**48**	**2.71**
		Protein modification and binding	9			
		Protein folding	8			
**7**	**Striated muscle contraction**	Contraction	26	**3.54**	**95**	**5.37**
		Calcium binding	10			
**8**	**Signal transduction**		8	**0.79**	**9**	**0.51**
**9**	**Structural constituent of cuticle**		14	**1.38**	**30**	**1.69**
**10**	**Cell growth, proliferation and adhesion**		9	**0.88**	**12**	**0.68**
**11**	**Ion Binding**		5	**0.49**	**5**	**0.28**
**12**	**Hypotetical protein**		10	**0.98**	**16**	**0.90**
**13**	**Mitochondrial genes**		14	**1.38**	**174**	**9.83**
**14**	**Ribosomal RNA gene (18S and 28S)**		14	**1.38**	**29**	**1.64**
**15**	**Without similarity**		708	**69.62**	**860**	**48.59**
TOTAL			**1017**	**100.00**	**1770**	**100.00**

The last four columns of the Table show respectively number and percentage of clusters and ESTs belonging to each functional category.

Other interesting krill transcripts that we were able to annotate are those involved in stress responses, proteolysis and immunoresponse (3.24%) like *Hsp90*, *chaperones*, *cathepsine L-like cysteine protease*, a lysosomal cysteine proteinase [22], *peptidyl-prolyl cis-trans isomerase A1 *(*cyclophilin *A1) and *peptidylprolyl isomerase B *(*cyclophilin *B). Cyclophilines are members of the immunophilin protein family, which play a role in immunoregulation and basic cellular processes involving protein folding and trafficking. ESTs with good similarity to hemocyanin are present in our collection: this protein has been recently reported to have antifungal and antiviral activities [23,24].

Some krill ESTs identify histone 2A (KRC00431) and histone 3.3A (KRC00024) indicating the presence of unexpected polyadenylated histone transcripts displaying the polyadenylation signal and tail. In vertebrates, these evolutionary conserved housekeeping mRNAs are not polyadenylated, and this has been related to the high turnover of these transcripts in the dividing cells. Interestingly, polyadenylated H2A and H3 histone sequences were detected also in the systematic sequencing of 3'-end cDNA libraries obtained from brain and kidney of channel catfish *Ictarulus punctatus *[25,26] and from various tissues (haemolymph, gills, digestive glands, mantles and adductor muscles) of the mussel *Mytilus galloprovincialis *[27]. The presence of polyadenylation signals in *E. superba *histone transcripts deserves a more detailed analysis. In fact, Eirin-Lopez *et al*. [28] have recently shown that all histone genes in the repetitive unit are characterized by two different mRNA termination signals in their 3' UTR: the typical stem-loop or hairpin-loop signal followed by a purine-rich element and a polyadenylation signal AATAAA located downstream to this last element. The presence of a double mRNA termination signal is unique to histone genes and common for other invertebrates such as *Chironomus thummi *[29], *D. melanogaster *[30], *Chaetopterus variopedatus *[31], *M. galloprovincialis *[32] and Crustacea [33]. Although in some invertebrates core histone transcripts (H2A, H2B, H3 and H4) include polyA tails, these sequences are among the most evolutionary conserved eukaryotic proteins [34].

### Transcriptional signature of *E. superba *tissues

Gene expression profiling depends on the functional specificity of cells composing different tissues. So, the systematic sequencing of EST from unbiased cDNA libraries is a suitable approach for analyzing the gene expression profile of a given tissue [35]. In fact, the frequency of a given EST in the cDNA library can be related to the relative abundance of the corresponding mRNA in the source tissue.

**Figure 2:**
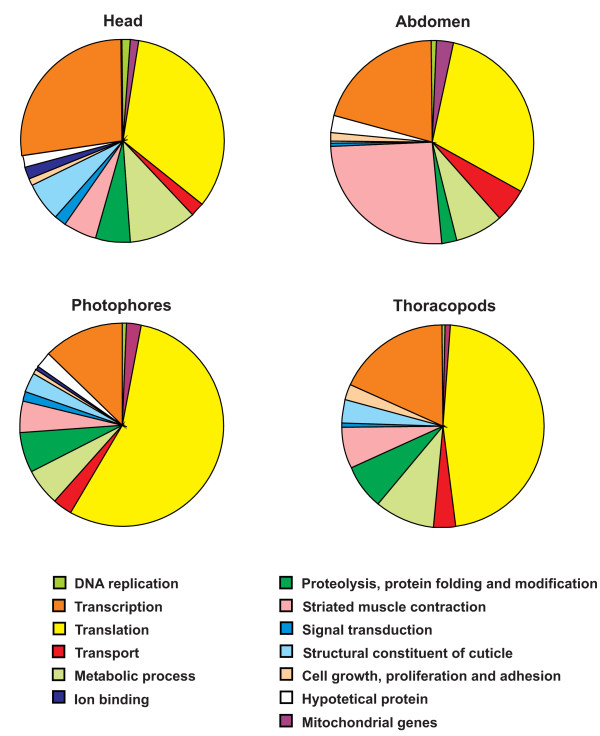
**Classification of the annotated ESTs for each library into different 13 functional categories**. Diagrams showing the proportion of each functional category in all four tissues. See Table 5 for more details.

To define tissue transcriptional signatures of *E. superba*, annotated ESTs obtained from the four tissue-specific cDNA libraries (head, abdomen, photophores, thoracopods) were separately grouped in 13 functional categories (Table 5) a further abundant category was created for those ESTs to which no function may be yet associated. Fig. 2 shows four different diagrams standing for ESTs distribution among functional categories in each cDNA library. The presence of highly represented functional categories is peculiar of strictly committed tissues such as abdomen and thoracopods in which transcripts involved in striated muscle contraction are very abundant (about 26% in abdomen and 7% in thoracopods). In the abdomen library, we were able to recognize the principal structural components of the sarcomeric contractile machinery (*myosin heavy chain*,* myosin light chain 1*, *myosin-2*, *actin*,* alpha-tubulin*,* tropomyosin*) and two subunits of the troponin complex (*troponin T*,* troponin I*), a key regulator of muscle contraction. About 10% of sequences produced from head and thoracopods libraries fall in functional categories related to metabolic processes (amino acid, fatty acid and carbohydrate metabolism). Interestingly, about 6% and 4% of ESTs respectively sequenced from head and thoracopods libraries identified structural constituents of cuticle (*arthrodial cuticle protein AMP16.3*,* arthrodial cuticle protein AMP1A*,* calcification-associated peptide-1 precursor*). This reflects the presence of cuticle traces in the head and thoracopods samples. In photophores and thoracopods transcripts displaying putative identity with ribosomal sequences are more abundant compared to other tissues (55% and 46%, respectively), indicating a relevant activity of the translation machinery.

**Table 5:** Classification of the annotated ESTs for each library.

**#**	**Process**	**Head**		**Abdomen**		**Photophores**		**Thoracopods**	
		**# ESTs**	**%**	**# ESTs**	**%**	**# ESTs**	**%**	**# ESTs**	**%**

1	**DNA replication**	3	1.43	2	0.90	2	0.88	1	0.45
2	**Transcription**	3	1.43	6	2.71	5	2.19	2	0.90
3	**Translation**	69	32.86	65	29.41	126	55.26	103	46.40
4	**Transport**	5	2.38	12	5.43	7	3.07	8	3.60
5	**Metabolic process**	22	10.48	16	7.24	14	6.14	21	9.46
6	**Proteolysis, protein folding and modification**	12	5.71	6	2.71	14	6.14	16	7.21
7	**Striated muscle contraction**	11	5.24	57	25.79	12	5.26	15	6.76
8	**Signal transduction**	4	1.90	1	0.45	3	1.32	1	0.45
9	**Structural constituent of cuticle**	13	6.19	1	0.45	7	3.07	9	4.05
10	**Cell growth, proliferation and adhesion**	2	0.95	3	1.36	2	0.88	5	2.25
11	**Ion Binding**	4	1.90	0	0.00	1	0.44	0	0.00
12	**Hypotetical protein**	4	1.90	6	2.71	6	2.63	0	0.00
13	**Mitochondrial genes**	58	27.62	46	20.81	29	12.72	41	18.47

	**Total**	**210**	100.00	**221**	100.00	**228**	100.00	**222**	100.0

Table showing the number and the percentage of ESTs in each functional category for each tissue specific library

We have also identified from the head cDNA library a novel opsin sequence (ID ESTs: KRC00735, KRC00802), a light-sensitive membrane-bound G protein-coupled receptors mediating the conversion of a photon of light into an electrochemical signal in the visual transduction cascade. In insects there are at least four main spectral classes: long-wavelenght-sensitive (LWS), middle-wavelenght-sensitive (MWS) and two short-wavelenght-sensitive (SWS) groups. The opsin sequences available for *E. superba *(GenBank accession no. DQ852576–DQ852580) show a spectral sensitivity with short wavelength (496–501 nm, λ_max _= 487) and cannot be aligned with our consensus [36].

Quantitative RT-PCR analysis was performed to quantify and validate the expression level of some genes presenting different EST countings in krill tissues. We selected ten genes (*compound eye opsin BCRH1*,*  myosin light chain*,* myosin heavy chain*, *arthrodial cuticle protein AMP16.3*,* tail muscle elongation factor 1 gamma*,* cellular retinoic acid/retinol binding protein*,* eukaryotic initiation factor 4A*,* transport protein SEC61 subunit gamma*,* chromodomain helicase DNA binding protein *and *voltage-dependent calcium channel*) representative of different levels of transcript abundance.

The housekeeping gene 18S rRNA was used as endogenous control. As reported in the Additional file 2, the expression values obtained with the quantitative RT-PCR for the tested transcripts were in agreement with the EST counting in the four libraries. In particular, we have demonstrated that the *compound eye opsin *is strongly expressed in head compared to other tissues and *myosin light chain *and *myosin heavy chain *are highly expressed in abdomen and thoracopods confirming their key role in the contractile machinery. Instead, the *eukaryotic initiation factor 4A *is expressed at about the same level in all tested tissues.

### Identification of microsatellite-containing ESTs

Among the 1,017 non-redundant sequences examined in this study, 41 (4%) consensus sequences containing ESTs were identified by using MISA software. Twelve of these consensus present 2 distinct simple sequence repeats interrupted by more than 100 bp for a total of 69 identified microsatellites (SSR). The majority of these sequences (72%) fall into the 3 bp repeat type class with a preponderance of GAA and GAT. After a manual inspection of redundancy, raw sequence, data quality and the presence of sufficient flanking sequences we designed 9 pairs of specific PCR primers. We obtained successful amplifications for 6 of these 9 pairs of primers. Assessment of polymorphism information content (PIC), observed and expected heterozygosity and other population genetics analysis will be performed in the near future. These markers will increase the currently available Euphausiacea SSR markers. In fact, only five microsatellite loci isolated from the northern krill *Meganyctiphanes norvegica *have been reported so far [37]. Since our novel microsatellite markers were developed on the basis of expressed sequences and they are presumably conserved across other Euphausiacea species, they could also be useful for comparative mapping and for a molecular approach to Antarctic krill ecology.

## Conclusion

Since genome sequencing and BAC libraries of Antarctic krill are not yet available, EST sequencing from randomly selected cDNA clones represents a powerful approach to identify large numbers of transcripts that could be used in gene expression and functional genomics studies [38]. The systematic sequencing of four cDNA libraries prepared from different *E. superba *tissues has allowed us to establish an EST database containing 1,017 unique sequences. Over 65% of the Antarctic krill sequences resulted in no BLAST matches with published sequences and they probably represent novel genes that could be functionally characterized. We have defined the transcriptional signatures of krill tissues and performed qRT-PCR to validate the level of expression of ten representative genes. All sequencing data have been deposited on the *E. superba *EST database available from our web site [16]. In addition, the EST collection is a potential source for the development of genetic markers including microsatellite and single nucleotide polymorphisms. Among the 1,017 unique sequences, 41 (4%) unique microsatellite containing ESTs were identified by using MISA software. Moreover, we have designed and successfully tested 6 pairs of specific PCR primers for microsatellite loci.

Our EST catalogue could provide a source for the design of microarray platform that will allow the study of the transcriptional responses of this abundant marine organism to environmental challenges [39].

## Methods

### Tissues samples, RNA extraction and quality control

Antarctic krill (*Euphausia superba*) were fished from the Ross Sea (longitude: 167°28'81" E – 179°54'68" W, latitude 68°40'54" S – 77°01"81" S) in the January 2004 during the XIX Italian Antarctic Expedition. Specimens were collected at different time of the day (01:00, 06:00, 10:00, 15:00, 18:00), over a complete 24-hour cycle. Samples were frozen at -40°C in RNA stabilization solution (RNA-later, Ambion). For each fishing, selected tissues (head including compound eyes and brain, abdomen, thoracopods and photophores) from five animals were dissected individually in RNA later ice solution (Ambion). After dissection, tissues were rapidly rinsed in sterile water, weighed, frozen in Trizol reagent (Invitrogen) and stored at -80°C. A large excess of Trizol (15 ml for 0.5–1.5 g. of sample) was used in order to prevent RNA degradation by endogenous RNAse. Frozen tissues were minced and homogenized for 3–5 min using an ultra-turrax-T8.01 blender (IKA-Werke). Total RNA was isolated using the Trizol reagent (Invitrogen) following the manufacturer's instruction and further purified with LiCl in order to remove glucidic contaminants. All RNA samples were checked for quality by capillary electrophoresis (RNA 6000 Nano LabChip, Agilent Bioanalyzer 2100, Agilent Technologies). For each tissue (head, abdomen, thoracopods and photophores), equal amounts of total RNA (2 μg) extracted from every collection were pooled.

### Construction of cDNA libraries

Five independent cDNA libraries, named K01 and K05 (head), K06 (abdomen), K07 (photophores) and K09 (thoracopods), were constructed.

**Figure 3:**
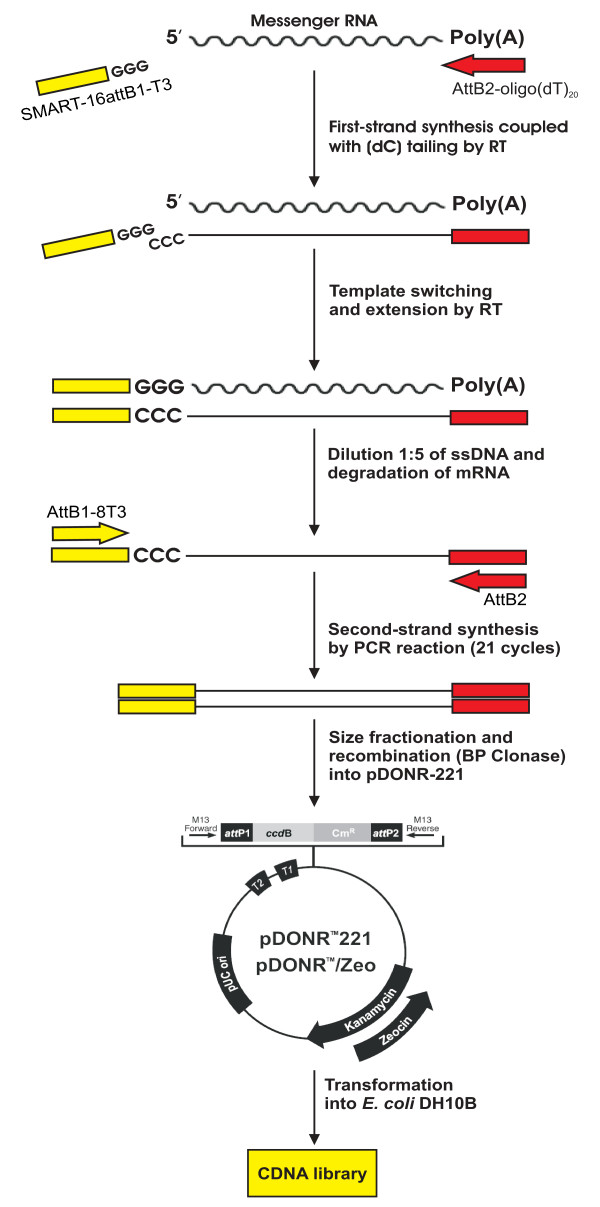
Schematic diagram of the method used for the construction of the cDNA libraries from different krill tissues. See the Methods for more details.

We have developed a new method using a combination of SMART protocol (Clontech), ensuring almost full-length cDNA, and Gateway technology (Invitrogen), allowing unidirectional cloning without enzymatic digestion. In this protocol, only fully-transcribed first strand cDNA (ss cDNA) is tagged with a short sequence complementary to a modified SMART oligo (Fig. 3). The SMART oligo sequence (SMART-16attB1-T3: 5'-TACAAAAAAGCAGGCTAATTAACCCTCACTAAAGGG-3') and the overhang of the oligo(dT) primer (5'-GGGGACCACTTTGTACAAGAAAGCTGGGCGGCCGC [dT]_20_VN-3') used for first strand synthesis include an attB1 and attB2 recombination site respectively.

First strand cDNA synthesis was performed from 1.5 μg of total RNA in a 15 μl reaction. Then, the reaction was then diluted 1:5 ratio and incubated at 72°C for 2 min. Second strand reaction mix was added to 1 μl of diluted first strand cDNA to give a final concentration of 1× BD Advantage 2 PCR reaction buffer (Clontech), 0.2 mM dNTPs, 120 nM primers (attB1-8T3: 5'-GGGGACAAGTTTGTACAAAAAAGCAGGCTAATTAACC-3' and attB2: 5'-GGGGACCACTTTGTACAAGAAAGCTGGG-3') and 1× of Advantage2 DNA polymerase mix (Clontech) in a volume of 50 μl. This second strand reaction mixture was incubated for 21 cycles of 15 sec 95°C, 30 sec 66°C and 3 min 68°C. Only those ss cDNAs having a SMART anchor sequence at the 5' end were used as template and exponentially amplified. The second strand reaction was glass fibre column purified and cDNA was size selected by Sepharose CL-4B SPUN COLUMN (GE Healthcare). The cDNA was inserted in the cloning vector by a recombination reaction performed at 25°C for 18 h with about 35 ng of attB-cDNA, 150 ng of pDONR221 (Invitrogen) and 2 μl of BP Clonase II (Invitrogen) in 10 μl final volume. 1/5 of the purified reaction was used to transform electrocompetent DH10B *E. coli *cells. Recombinant colonies were selected on agar SOB medium plus kanamicin. Individual library colonies were arrayed by manual picking on 96 well plates in liquid selective SOB medium plus 7.5% of glycerol for independent growth [40].

### DNA sequencing

After lysis of the bacterial colonies, cDNA inserts were directly amplified with universal primers M13 forward (5'-TGTAAAACGACGGCCAGTCTTA-3') and M13 reverse (5'-CAGGAAACAGCTATGACCATGT-3'). The polymerase chain reaction (PCR) profile consisted of: (1) initial denaturation for 5 min at 95°C, (2) 35 cycles of 40-s denaturation at 95°C, 40-s annealing at 60°C and 1-min elongation at 72°C and (3) final extension for 5 min at 72°C; samples with a size over 0.5 Kb were selected for sequencing. Single pass DNA sequencing from plasmids was performed by using the vector specific primer attB1_seq (5'-CTTTGTACAAAAAAGCAGGCT-3') and a modified Sanger dideoxy terminator cycle sequencing chemistry, the ABI BigDye kit version 3.1, on a ABI 3730 48-capillary sequencer and 36 cm capillaries (Sequencing Service of Max-Plank Institute for Molecular Genetics, Berlin, Germany).

### Computer Management of Data

Trace2dbest and Partigene [41] were used to process chromatograms, clusterize sequences, and build an annotation database. Trace2dbest extracts sequences and quality information from traces (Phred algorithm), removes vector contamination and poly(A), and performs the trimming of low quality sequences. Sequences shorter than 100 bp were discarded. Partigene reads all sequence files and performs an assembling process in two step: 1) CLOBB software [42] clusterizes sequences on the basis of BLAST similarity; 2) Phrap [43] makes a consensus from each cluster.

Each consensus, converted in FASTA format, was searched locally in nucleotides database, downloaded from NCBI [44] and UniProtKB database [45], using Blast-N and Blast-X, respectively. First 10 HSPs (High Scoring Pair) from each blast result were collected and stored in a local PostgreSQL table, as a collection of automatic annotations.

Each cluster annotation in our database was further manually examined to assign the best describing text to the correspondent cluster: matches with expectations values greater than e^-10 ^for protein (Blast-X) and e-^40 ^for nucleotide (Blast-N) were considered as poorly informative. Moreover, for each UniProt ID, taken from Blast-X description field, we associated specific Gene Ontology annotation, that integrates information about process, function, and component. Clusters, consensus and related similarity and gene ontology searches were electronically organized and stored in a dedicated PostgreSQL database.

### Identification of microsatellite containing ESTs

The unique sequences were screened for microsatellites by using the MISA software [46]. Only di-, tri-, tetra-, penta- and esanucleotide repeats were targeted, since mononucleotide repeats are not useful for mapping or population genetics due to difficulties in their genotyping. Strings of oligonucleotide sequences were used to search for microsatellites: 6 repeats for dinucleotide; 5 repeats for trinucleotide; 5 repeats for tetranucleotide, pentanucleotide and esanucleotide. Primers were designed for the flanking regions of the SSR using a web-based software, "Primer3" [47], and based on the criteria of 50% GC content, a minimum melting temperature of 55°C, and absence of secondary structure. Primers ranged from 18–27 nucleotides in length and amplified products of 150–390 bp. The primers were synthesized with a 5'-KS-tail (KS sequence: 5'-cgaggtcgacggtatcg-3') allowing to amplify microsatellite alleles in combination with a 5'-fluorescent-labeled KS primer [48].

### Quantitative RT-PCR

Quantitative RT-PCR was conducted for some genes using the same tissues tested (head, abdomen, thoracopods and photophores) to confirm the integrity and robustness of EST sequencing.

Three μg of total RNA from each tissue was used to perform three independent cDNA syntheses in a final volume of 10 μl, using random decamers and SuperScript II reverse transcriptase (Invitrogen). 1 μl aliquot of diluted first-strand cDNA was PCR amplified in 10 μl volume using SYBR Green chemistry, according to the manufacturer's recommendations (Finnenzymes). Gene-specific primers were designed using Primer Express^® ^Software (Applera) to amplify fragments of 120–180 bp in length, close to the 3'-end of the transcript. To avoid the amplification of contaminant genomic DNA, we treated total RNA samples with DNase I (Qiagen). The dissociation curve was used to confirm the specificity of the amplicon. PCR reactions were performed in a 7500 Real-Time PCR System (Applied Biosystems). Thermal cycling conditions were as follows: 15 min denaturation at 95°C; followed by 40 cycles of 30 sec denaturation step at 95°C, annealing and elongation steps for 1 min each at 60°C and a final 3 min elongation at 72°C. To evaluate differences in gene expression a relative quantification method was chosen where the expression of the target gene is standardized by a non-regulated reference gene; consequently, three replicates of each sample and endogenous control were amplified. 18S rRNA was used as an endogenous control because the level of rRNA remains essentially constant from sample to sample (QuantumRNA™ 18S Internal Standards, Ambion). To calculate the relative expression ratio, the 2^-ΔΔCt ^(RQ, relative quantification) method implemented in the 7500 Real Time PCR System software [49] was used. This method determines the change in expression of a nucleic acid sequence (target) in a test sample relative to the same sequence in a calibrator sample. In our experiments, the expression of ten targets was tested (*compound eye opsin BCRH1*, *myosin light chain*, *myosin heavy chain*,* arthrodial cuticle protein AMP16.3*,* tail muscle elongation factor 1 gamma*,* cellular retinoic acid/retinol binding protein*,* eukaryotic initiation factor 4A*,* transport protein SEC61 subunit gamma*, *chromodomain helicase DNA binding protein and voltage-dependent calcium channel*) which displayed differential expression in the head, thoracopods and photophores, compared with the abdomen.

## Availability and requirements

Project name: Systematic sequencing of mRNA from the Antarctic krill (*Euphausia superba*);

Project home page: ;

Operating system(s): Debian GNU/Linux;

Programming language: PHP;

Licence: none;

Any restrictions to use by non-academics: none.

## Authors' contributions

CDP performed total RNA sample preparation, construction and systematic sequencing of the cDNA libraries, annotation of ESTs, qRT-PCR and drafted the manuscript. CB conceived the study, carried out ESTs analysis and drafted the manuscript. GMM and GR participated in systematic sequencing of cDNA libraries, in design of the study and revision of the manuscript. FB performed bioinformatic analysis of cDNA libraries sequence data, clustering of ESTs and annotation of ESTs and identification of microsatellite containing ESTs. BDN and AP participated in development of cDNA libraries production method and identification of microsatellite containing ESTs. GL supervised the study, participating in the design and coordination of the work, the interpretation of the results and revision of the manuscript. RC supervised the study, participating in the design and coordination of the work, the interpretation of data and manuscript writing. All Authors read and approved the final version of the manuscript declaring that they have no potential conflicts of interests.

## Supplementary Material

Additional file 1 This table lists 309 non-redundant sequences identifying known *E. superba *genes or sequences showing significant similarity with genes from arthropods and other species. These transcripts have been grouped into 13 different functional categories. Click here for file

Additional file 2 Quantitative RT-PCR validation. Forward and reverse primer pairs for ten tested transcripts are detailed at the top. Relative expression values resulting from the quantitative real-time PCR are in the middle and related data plots are also reported. For each transcriptional profiling we have associated the EST counting obtained from the cDNA libraries systematic sequencing. Click here for file
