# Supplementary material for: Systematic sequencing of mRNA from the Antarctic krill (Euphausia superba) and first tissue specific transcriptional signature
Source: BMC Genomics. 2008 Jan 28;9:45. doi: 10.1186/1471-2164-9-45 (PMC2270838; doi:10.1186/1471-2164-9-45)
Supplement: Additional file 1 — This table lists 309 non-redundant sequences identifying known E. superba genes or sequences showing significant similarity with genes from arthropods and other species. These transcripts have been grouped into 13 different functional categories. [file 1471-2164-9-45-S1.PDF]

Additional Table 1

1. DNA replication & binding

Cluster ID	# EST	e-value	Program	Putative identity	Organism	Protein ID	GO Biological Process
KRC00431	1	3E-35	blast X	Histone 2A Z variant	*B. villosa*	Q8MVI7	chrom. organization and biogenesis (GO:0007001)
KRC00024	1	2E-69	blast X	Similar to Drosophila melanogaster His3.3A (Fragment)	*D. yakuba*	Q6XIQ5	chrom. organization and biogenesis (GO:0007001)
KRC00336	1	1E-25	blast X	Cisplatin resistance-associated overexpressed protein	*P. pygmaeus*	Q5R8W6	DNA binding (GO:0003677)
KRC00017	1	8E-10	blast X	Replication factor C subunit 4	*M. musculus*	Q99J62	DNA replication (GO:0006260)
KRC00723	1	2E-51	blast X	DNA replication licensing factor MCM5	*A. aegypti*	Q17H38	DNA replication initiation (GO:0006270)
KRC00275	1	1E-14	blast X	Topoisomerase III alpha	*G. gallus*	Q4W5X0	DNA topological change (GO:0006265)
KRC01009	1	7E-22	blast X	RBM25 protein	*H. sapiens*	Q2TA72	nucleotide binding (GO:0000166)
KRC00061	1	9E-14	blast X	RNA binding motif protein 28 variant (Fragment)	*H. sapiens*	Q53H65	nucleotide binding (GO:0000166)

2. Transcription

Cluster ID	# EST	e-value	Program	Putative identity	Organism	Protein ID	GO Biological Process
KRC00007	1	2E-38	blast X	Nucleosome remodeling factor subunit NURF301	*D. melanogaster*	Q9W0T1	chromatin remodeling (GO:0006338)
KRC00018	1	2E-14	blast X	Lipopolysaccharide-induced tumor necrosis factor-alpha	*X. Tropicalis*	Q6P828	regulation of transcription (GO:0006355)
KRC00084	1	4E-24	blast X	Forkhead transcription factor N2/3 (Fragment)	*S. purpuratus*	Q2V887	regulation of transcription (GO:0006355)
KRC00095	1	2E-32	blast X	Pyrophosphate phospho-hydrolase (PPase)	*D. melanogaster*	O77460	chromatin remodeling (GO:0006338)
KRC00133	1	4E-32	blast X	Regulator of sex-limitation candidate 1	*M. musculus*	Q7M6W8	regulation of transcription (GO:0006355)
KRC00236	1	2E-19	blast X	Nfxl1 protein	*M. musculus*	Q8R217	regulation of transcription (GO:0006355)
KRC00295	1	4E-19	blast X	Cre binding protein-like 2 (CREBL2 protein)	*H. sapiens*	O60519	regulation of transcription (GO:0006355)
KRC00339	2	3E-10	blast X	Transcription elongation factor A (SII), 2	*B. rerio*	Q7ZUY7	transcription (GO:0006350)
KRC00402	1	6E-58	blast X	Chromodomain helicase DNA binding protein	*A. aegypti*	Q16K27	chromatin assembly or disassembly (GO:0006333)
KRC00497	1	2E-10	blast X	Gypsy Danio rerio 1 polyprotein	*B. rerio*	Q8JIX4	regulation of transcription (GO:0006355)
KRC00533	1	3E-20	blast X	Chmadrin (Short type)	*P. tridactylus*	Q9XS53	chromatin assembly or disassembly (GO:0006333)
KRC00747	1	4E-11	blast X	DNA-directed RNA polymerase II 140 kDa polypeptide	*D. melanogaster*	P08266	transcription (GO:0006350)
KRC00833	1	3E-15	blast X	Chromobox-like protein 1 (Predicted protein)	*A. aegypti*	Q1HQQ4	chromatin assembly or disassembly (GO:0006333)
KRC00989	1	1E-25	blast X	Zinc finger protein 760	*M. musculus*	Q5U4A6	regulation of transcription (GO:0006355)
KRC01027	1	7E-11	blast X	TAF5 RNA polymerase II, TBP associated factor	*B. rerio*	Q32PW4	regulation of transcription (GO:0045449)

3. Translation

Cluster ID	# EST	e-value	Program	Putative identity	Organism	Protein ID	GO Biological Process
KRC00010	16	7E-23	blast X	60S acidic ribosomal protein P1 (EL12`/ EL12`-P)	*A. salina*	P02402	translation (GO:0006412)
KRC00020	31	1E-30	blast X	Ribosomal protein S25	*I. scapularis*	Q4PM82	translation (GO:0006412)
KRC00021	2	1E-11	blast X	S10e ribosomal protein	*C.granulatus*	Q6EUZ6	translation (GO:0006412)
KRC00025	6	9E-71	blast X	40S ribosomal protein S6	*D. melanogaster*	P29327	translation (GO:0006412)
KRC00043	7	9E-66	blast X	Elongation factor 1-alpha (EF-1-alpha)	*R. racemosus*	P14864	translational elongation (GO:0006414)
KRC00051	9	2E-53	blast X	40S ribosomal protein S14	*P. clarkii*	P48855	translation (GO:0006412)
KRC00052	1	2E-18	blast X	40S ribosomal protein S21	*C. capitata*	Q8I7N7	translation (GO:0006412)

Cluster ID	# EST	e-value	Program	Putative identity	Organism	Protein ID	GO Biological Process
KRC00054	13	3E-12	blast X	Ribosomal protein P2 (60S acidic ribosomal protein P2)	*B. mori*	Q5UAT9	translation (GO:0006412)
KRC00056	1	1E-15	blast X	Putative elongation factor 1 beta`	*D. citri*	Q0PXV3	translational elongation (GO:0006414)
KRC00058	1	5E-14	blast X	40S ribosomal protein S28	*R. norvegicus*	P62859	translation (GO:0006412)
KRC00073	5	1E-52	blast X	40S ribosomal protein S12	*D. variabilis*	Q86FP6	translation (GO:0006412)
KRC00076	1	7E-14	blast X	Putative elongation factor 1 beta`	*D. citri*	Q0PXV3	translational elongation (GO:0006414)
KRC00077	1	2E-34	blast X	Ribosomal protein S26	*P. xylostella*	Q6F480	translation (GO:0006412)
KRC00089	1	2E-24	blast X	Putative translation elongation factor 2	*T. citricida*	Q5XUB4	translational elongation (GO:0006414)
KRC00099	3	6E-31	blast X	Ribosomal protein L13	*L. testaceipes*	Q56FE7	translation (GO:0006412)
KRC00105	6	2E-85	blast X	Ribosomal protein L7e	*B. lunatus*	Q4GXM3	translation (GO:0006412)
KRC00109	3	3E-28	blast X	60S ribosomal protein L36	*H. sapiens*	Q6FIG1	translation (GO:0006412)
KRC00111	2	1E-112	blast X	Ribosomal protein L8	*P. vannamei*	Q2I3E8	translation (GO:0006412)
KRC00116	2	6E-42	blast X	40S ribosomal protein S23 (Ribosomal protein S12, putative)	*A. aegypti*	Q1HRM5	translation (GO:0006412)
KRC00126	6	1E-104	blast X	Ribosomal protein L4	*X. tropicalis*	Q28G60	translation (GO:0006412)
KRC00129	3	3E-58	blast X	60S ribosomal protein L5	*H. annuus*	O65353	translation (GO:0006412)
KRC00131	6	1E-100	blast X	Ribosomal protein S4e	*C. granulatus*	Q4GXU6	translation (GO:0006412)
KRC00157	1	1E-46	blast X	40S ribosomal protein S15a	*D. yakuba*	Q6XIM8	translation (GO:0006412)
KRC00167	3	3E-34	blast X	60S ribosomal protein L15	*O. limosus*	Q9XYC2	translation (GO:0006412)
KRC00197	5	2E-61	blast X	40S ribosomal protein S13	*R. norvegicus*	P62278	translation (GO:0006412)
KRC00214	8	6E-44	blast X	60S ribosomal protein L23a	*R. norvegicus*	P62752	translation (GO:0006412)
KRC00227	2	1E-26	blast N	Ribosomal protein L3 (RpL3) mRNA	*B. mori*	AY769270.1	translation (GO:0006412)
KRC00231	2	2E-70	blast X	40S ribosomal protein S8	*A. mellifera*	O76756	translation (GO:0006412)
KRC00238	31	6E-37	blast N	Ribosomal protein L22 mRNA	*A. irradians*	AF526199.1	translation (GO:0006412)
KRC00258	1	6E-35	blast N	Ribosomal protein L10 mRNA	*C. sapidus*	AY822650.1	translation (GO:0006412)
KRC00269	10	5E-66	blast X	40S ribosomal protein S3	*R. norvegicus*	P62909	translation (GO:0006412)
KRC00272	7	2E-40	blast N	40S ribosomal protein S5 mRNA, partial cds	*P. major*	AY190724.1	translation (GO:0006412)
KRC00294	8	1E-35	blast N	Ribosomal protein L23-like protein mRNA	*C. quadricarinatus*	AY642686.1|	translation (GO:0006412)
KRC00299	15	1E-34	blast X	Ribosomal protein L37A-like	*A. pisum*	Q201W6	translation (GO:0006412)
KRC00303	13	3E-41	blast X	60S ribosomal protein L24	*S. frugiperda*	Q962T5	translation (GO:0006412)
KRC00307	1	3E-97	blast X	Tail muscle elongation factor 1 gamma	*P. clarkii*	Q197G1	translational elongation (GO:0006414)
KRC00312	4	2E-55	blast N	S2 ribosomal protein (S2) gene	*M. poutassou*	AY292480.1	translation (GO:0006412)
KRC00319	2	2E-58	blast X	Tail muscle elongation factor 1 gamma	*P. clarkii*	Q197G1	translational elongation (GO:0006414)
KRC00325	6	1E-86	blast X	Elongation factor 2 (Fragment)	*C. idella*	Q8AY86	translational elongation (GO:0006414)
KRC00326	4	3E-46	blast X	40S ribosomal protein S17	*S. scrofa*	Q6QAP7	translation (GO:0006412)
KRC00332	6	3E-17	blast X	Ribosomal protein L35	*C. parallela*	Q8MUR3	translation (GO:0006412)
KRC00340	2	1E-55	blast X	Ribosomal protein L19e	*C. campestris*	Q4GXG0	translation (GO:0006412)
KRC00358	2	2E-44	blast X	Ribosomal protein L7Ae	*S. laticollis*	Q4GXL6	translation (GO:0006412)
KRC00360	2	3E-39	blast X	Ribosomal protein L6e (Fragment)	*Georissus sp.*	Q4GXM8	translation (GO:0006412)
KRC00363	5	8E-55	blast X	Ribosomal protein S15	*B. belcheri*	Q6IV90	translation (GO:0006412)
KRC00364	5	5E-45	blast X	Ribosomal protein L31 isoform A	*L. testaceipes*	Q56FI5	translation (GO:0006412)
KRC00386	3	2E-35	blast X	60S ribosomal protein L13A	*M. edulis*	Q3HNK3	translation (GO:0006412)

Cluster ID	# EST	e-value	Program	Putative identity	Organism	Protein ID	GO Biological Process
KRC00403	2	1E-39	blast X	Ribosomal protein L35Ae	*C. granulatus*	Q4GXA3	translation (GO:0006412)
KRC00404	5	2E-75	blast X	Ribosomal protein L10a isoform A	*L. testaceipes*	Q56FD8	translation (GO:0006412)
KRC00406	3	4E-68	blast X	Ribosomal protein L9e	*C. litorea*	Q4GXL0	translation (GO:0006412)
KRC00419	9	2E-31	blast X	Putative ribosomal protein L34	*D. citri*	Q0PXZ3	translation (GO:0006412)
KRC00425	7	6E-72	blast X	40S ribosomal protein S7	*M. musculus*	P62082	translation (GO:0006412)
KRC00442	8	6E-50	blast X	Ribosomal protein L32	*B. belcheri*	Q86QS0	translation (GO:0006412)
KRC00455	5	4E-28	blast N	40S ribosomal protein S21 mRNA	*I. punctatus*	AF402830.1	translation (GO:0006412)
KRC00459	1	1E-27	blast X	60S ribosomal protein L14	*B. rerio*	Q6DRN7	translation (GO:0006412)
KRC00491	2	3E-59	blast X	60S ribosomal protein L18a	*R. norvegicus*	P62718	translation (GO:0006412)
KRC00494	7	4E-97	blast N	Ribosomal protein S18 mRNA, partial cds	*C. destructor*	AY014897.1	translation (GO:0006412)
KRC00502	1	8E-24	blast X	60S ribosomal protein L37	*S. frugiperda*	Q962S7	translation (GO:0006412)
KRC00511	1	5E-27	blast N	Ubiquitin/ribosomal protein S27a fusion protein (Ubf80) mRNA	*B. belcheri*	AY066029.1	translation (GO:0006412)
KRC00530	2	1E-40	blast X	Ribosomal protein L44e	*Eucinetus sp.*	Q4GX69	translation (GO:0006412)
KRC00539	6	1E-117	blast N	Ribosomal protein L8 mRNA	*L. vannamei*	DQ316258.1	translation (GO:0006412)
KRC00541	3	2E-61	blast X	Putative ribosomal protein L17	*D. citri.*	Q0PXV9	translation (GO:0006412)
KRC00543	1	4E-49	blast X	Ribosomal protein S11	*G. gallus*	Q98TH5	translation (GO:0006412)
KRC00552	1	2E-21	blast X	40S ribosomal protein S29	*D. melanogaster*	Q9VH69	translation (GO:0006412)
KRC00577	3	2E-78	blast X	60S ribosomal protein L11	*A. albopictus*	Q5MIR0	translation (GO:0006412)
KRC00634	1	4E-33	blast X	Protein translation factor SUI1 homolog	*A. gambiae*	P42678	translational initiation (GO:0006413)
KRC00664	2	3E-54	blast X	Ribosomal protein L7Ae	*M. quadripustulatus*	Q4GXL7	translation (GO:0006412)
KRC00665	1	2E-51	blast X	Ribosomal protein L30	*A. irradians*	Q8ITC5	translation (GO:0006412)
KRC00714	2	9E-25	blast X	Ribosomal protein L22	*B. belcheri*	Q8ISQ5	translation (GO:0006412)
KRC00733	1	6E-21	blast X	60S ribosomal protein L39	*A. aegypti*	Q1HRB5	translation (GO:0006412)
KRC00760	1	1E-25	blast X	Basic leucine zipper and W2 domains 2	*M. musculus*	Q91VK1	translational initiation (GO:0006413)
KRC00786	2	1E-112	blast X	Eukaryotic initiation factor 4A	*C. sapidus*	Q0PZI5	translational initiation (GO:0006413)
KRC00789	2	4E-41	blast X	40S ribosomal protein S24	*F. rubripes*	O42387	translation (GO:0006412)
KRC00857	1	6E-24	blast X	60S ribosomal protein L27	*A. monolakensis*	Q09JK1	translation (GO:0006412)
KRC00879	1	7E-20	blast X	Ribosomal protein LP1	*A. monolakensis*	Q09JS1	translation (GO:0006412)
KRC00934	2	4E-64	blast X	40S ribosomal protein S16	*H. fossilis*	Q98TR7	translation (GO:0006412)
KRC00967	1	1E-15	blast X	Ribosomal protein L38	*P. xylostella*	Q6F450	translation (GO:0006412)
KRC00973	1	2E-20	blastX	beta-keto adipate succinyl CoA transferase	*P. putida*	Q16T93	translational termination (GO:0006415)
KRC00999	1	2E-10	blast X	60S ribosomal protein L39	*A. aegypti*	Q1HRB5	translation (GO:0006412)
KRC01018	1	5E-61	blast X	Ribosomal protein L26	*P. monodon*	Q5QDM7	translation (GO:0006412)
KRC01039	1	2E-19	blast X	Eukaryotic translation initiation factor 3 subunit 5 epsilon-like	*I. scapularis*	Q4PMC7	translational initiation (GO:0006413)
KRC01056	1	2E-25	blast X	60S acidic ribosomal protein P0	*S. frugiperda*	Q8WQJ2	translation (GO:0006412)

4. Transport

Cluster ID	# EST	e-value	Program	Putative identity	Organism	Protein ID	GO Biological Process
KRC00112	4	1E-43	blast X	ATP/ADP translocase	*P. leniusculus*	Q0GBZ2	transport (GO:0006810)
KRC00115	1	4E-12	blast X	Putative ubiquinol-cytochrome c reductase complex	*G. atropunctata*	Q1W286	electron transport (GO:0006118)
KRC00117	4	7E-27	blast X	Cytochrome c oxidase subunit VIa/COX13	*A. aegypti*	Q1HRM4	electron transport (GO:0006118)
KRC00136	1	2E-20	blast X	Mitochondrial import inner membrane translocase subunit Tim8	*D. melanogaster*	Q9Y1A3	transport (GO:0006810)
KRC00150	1	1E-12	blast X	Cytochrome oxidase subunit VIIc	*I. pacificus*	Q6B866	electron transport (GO:0006118)
KRC00160	1	4E-41	blast X	Voltage-dependent calcium channel (Fragment)	*A. aegypti*	Q16PI9	calcium ion transport (GO:0006816)
KRC00172	1	6E-31	blast X	Sec63 protein (Fragment)	*M. musculus*	Q8K2U5	intracellular protein transport (GO:0006886)
KRC00208	1	4E-26	blast X	Protein transport protein SEC61 subunit gamma	*C. intestinalis*	Q8I7D9	intracellular protein transport (GO:0006886)
KRC00222	1	1E-27	blast X	Cytochrome c oxidase polypeptide VIb (Fragment)	*D. mauritiana*	Q7YZE2	electron transport (GO:0006118)
KRC00263	2	6E-12	blast X	Transport protein SEC61 beta subunit	*A. aegypti*	Q1HR43	transport (GO:0006810)
KRC00281	1	9E-38	blast X	Clathrin heavy chain	*D. melanogaster*	P29742	intracellular protein transport (GO:0006886)
KRC00338	1	1E-31	blast X	Cellular retinoic acid/retinol binding protein	*M. ensis*	Q8WR15	transport (GO:0006810)
KRC00416	1	1E-21	blast X	Multidrug resistance protein 2 (Atp-binding cassette protein c)	*A. aegypti*	Q17BX9	transport (GO:0006810)
KRC00493	3	9E-57	blast X	Plasma membrane calcium-transporting atpase 3 (Pmca3)	*A. aegypti*	Q179M3	calcium ion transport (GO:0006816)
KRC00499	2	1E-18	blast X	Cytochrome c oxidase subunit IV	*B. mori*	Q1HQ98	electron transport (GO:0006118)
KRC00519	3	4E-45	blast X	NADH:ubiquinone dehydrogenase, putative	*A. aegypti*	Q17M02	electron transport (GO:0006118)
KRC00589	1	6E-24	blast X	Hemocyanin	*C. scammoni*	Q283K7	transport (GO:0006810)
KRC00911	1	4E-47	blast X	SEC13-related protein (SEC13-like protein 1)	*H. sapiens*	P55735	intracellular protein transport (GO:0006886)
KRC00916	1	1E-19	blast X	ATP/ADP translocase	*P. leniusculus*	Q0GBZ2	transport (GO:0006810)
KRC00985	1	1E-79	blast X	CG2014-PA	*D. melanogaster*	Q9VAK5	electron transport (GO:0006118)

5. Metabolic Process

Cluster ID	# EST	e-value	Program	Putative identity	Organism	Protein ID	GO Biological Process
KRC00623	1	7E-27	blast X	Glyoxylate reductase/hydroxypyruvate reductase	*B. mori*	Q1HPN4	amino acid metabolic process (GO:0006520)
KRC00927	1	6E-34	blast X	Cystathionase (Cystathionine gamma-lyase)	*H. sapiens*	Q53Y79	amino acid metabolic process (GO:0006520)
KRC00414	3	3E-20	blast X	H+ transporting ATP synthase subunit g	*B. mori*	Q1HQ25	ATP synt. coupled proton transport (GO:0015986)
KRC00602	1	5E-43	blast X	Vacuolar ATP synthase subunit h	*A. aegypti*	Q0IF50	ATP synt. coupled proton transport (GO:0015986)
KRC00929	1	2E-30	blast X	Putative ATP synthase oligomycin sensitivity conferral protein	*G. atropunctata*	Q1W2B6	ATP synt. coupled proton transport (GO:0015986)
KRC00512	5	4E-18	blast X	TP lipid-binding protein like protein (Fragment)	*P. japonicus*	Q8TA60	ATP synt. coupled proton transport (GO:0015986)
KRC00764	1	4E-26	blast X	Nucleotide binding protein 2	*R. norvegicus*	Q68FS1	ATP binding (GO:000552)
KRC00027	1	2E-11	blast X	Glutathione S-transferase M	*B. rerio*	Q6PFJ6	carbohydrate metabolic process (GO:0005975)
KRC00042	4	1E-151	blast X	Glyceraldehyde-3-phosphate dehydrogenase (GAPDH)	*P. versicolor*	P56649	carbohydrate metabolic process (GO:0005975)
KRC00093	1	1E-11	blast X	Isocitrate dehydrogenase	*C. gigas*	Q5QGY7	carbohydrate metabolic process (GO:0005975)
KRC00130	2	5E-80	blast X	Glyceraldehyde-3-phosphate dehydrogenase (GAPDH)	*H. americanus*	P00357	carbohydrate metabolic process (GO:0005975)
KRC00164	2	2E-13	blast X	Fructose-bisphosphate aldolase 2 (Aldolase CE-2)	*C. elegans.*	P46563	carbohydrate metabolic process (GO:0005975)
KRC00578	6	6E-17	blast X	Beta-N-acetylglucosaminidase	*C. fumiferana*	Q52H16	carbohydrate metabolic process (GO:0005975)
KRC00682	1	8E-11	blast X	Beta-hexosaminidase	*A. aegypti*	Q17C82	carbohydrate metabolic process (GO:0005975)
KRC00942	1	4E-30	blast X	Phosphoglycerate kinase 1	*B. rerio*	Q7ZV29	carbohydrate metabolic process (GO:0005975)

Cluster ID	# EST	e-value	Program	Putative identity	Organism	Protein ID	GO Biological Process
KRC00958	1	2E-27	blast X	Thioredoxin-1 (Fragment)	*M. cyprius*	Q685Y5	carbohydrate metabolic process (GO:0005975)
KRC00640	1	1E-15	blast X	Glutathione-requiring prostaglandin D synthase	*G. gallus*	O73888	fatty acid biosynthetic process (GO:0006633)
KRC00871	2	7E-33	blast X	Methylglutaconyl-CoA hydratase, putative	*A. aegypti*	Q0IEM7	fatty acid biosynthetic process (GO:0006633)
KRC00953	1	2E-09	blast X	5`-AMP-activated protein kinase subunit beta-1 (AMPKb)	*S. scrofa*	P80387	fatty acid biosynthetic process (GO:0006633)
KRC00706	1	3E-31	blast X	Histone deacetylase 3	*X. tropicalis*	Q28DV3	histone deacetylation (GO:0016575)
KRC00559	1	2E-35	blast X	Aspartate racemase	*A. broughtonii*	Q2L695	metabolic process (GO:0008152)
KRC00977	1	5E-23	blast X	Sterol carrier protein-2, putative	*A. aegypti*	Q17HG1	metabolic process (GO:0008152)
KRC01002	1	1E-66	blast X	ATP-citrate synthase	*A. aegypti*	Q17D87	metabolic process (GO:0008152)
KRC00504	5	2E-60	blast X	Nucleoside diphosphate kinase B (NDK B)	*R. norvegicus*	P19804	nucleotide metabolic process (GO:0009117)
KRC01071	1	3E-35	blast X	Adenosylhomocysteinase	*B. belcheri*	Q6WN55	one-carbon metabolic process (GO:0006730)
KRC00473	1	1E-20	blast X	Ethanolamine-phosphate cytidylyltransferase	*M. musculus*	Q922E4	phospholipid metabolic process (GO:0006644)
KRC01040	1	2E-29	blast X	Nucleoside diphosphate kinase	*O. mykiss*	Q804Y0	pyrimidine ribonucleoside biosynthesis (GO:0009209)
KRC00628	2	3E-21	blast X	Selenium dependent salivary glutathione peroxidase	*I. scapularis*	Q4PMF0	response to oxidative stress (GO:0006979)
KRC00187	1	2E-63	blast X	Superoxide dismutase 2, mitochondrial	*B. rerio*	Q6P980	superoxide metabolic process (GO:0006801)
KRC00022	6	1E-147	blast X	Receptor for activated protein kinase C RACK isoform 1	*B. mori*	Q2F5M4	kinase activity (GO:0016301)
KRC00088	3	6E-19	blast X	NADPH-dependent FMN reductase	*A. ehrlichei*	Q0A533	carbohydrate metabolic process (GO:0005975)
KRC00137	1	3E-61	blast X	Glutathione S-transferase	*A. gambiae*	O76482	transferase activity (GO:0016740)
KRC00189	1	5E-29	blast X	Lysophospholipase-like protein 1	*M. musculus*	Q3UFF7	hydrolase activity (GO:0016787)
KRC00253	4	4E-30	blastX	Creatine kinase, mitochondrial 1	*B. rerio*	Q7ZUN7	kinase activity (GO:0016301)
KRC00528	1	8E-21	blast X	C-type lectin (Hypothetical protein)	*A. aegypti*	Q1HRD1	sugar binding (GO:0005529)
KRC00576	1	2E-19	blast X	Acyl-CoA-binding domain-containing protein 6	*B. rerio*	Q4V8X4	acyl-CoA binding (GO:0000062)
KRC00590	1	1E-21	blast X	Mitochondrial ATP synthase F chain-like	*A. pisum*	Q201W3	carbohydrate metabolic process (GO:0005975)
KRC00771	1	5E-20	blast X	Creatine kinase, mitochondrial 2 (Sarcomeric)	*B. rerio*	Q6PC86	kinase activity (GO:0016301)
KRC00905	1	4E-23	blast X	Phosphoethanolamine N-methyltransferase	*A. tripolium*	Q84SA4	methyltransferase activity (GO:0008168)
KRC00302	1	9E-43	blast X	Bifunctional 3`-phosphoadenosine 5`-phosphosulfate synthet.	*U. caupo*	Q27128	sulfate assimilation (GO:0000103)
KRC00983	1	5E-28	blast X	Mannose-binding protein	*P. leniusculus*	Q56P33	sugar binding (GO:0005529)

6. Proteolysis, protein folding and modification

Cluster ID	# EST	e-value	Program	Putative identity	Organism	Protein ID	GO Biological Process
KRC00468	1	4E-23	blast X	Cathepsin A	*B. belcheri*	Q6WLC2	proteolysis (GO:0006508)
KRC00785	1	4E-46	blast X	Cathepsin C	*H. sapiens*	Q71E76	proteolysis (GO:0006508)
KRC00647	3	2E-20	blast X	Fibrinolytic enzyme (Fragment)	*E. sinensis*	Q27J28	proteolysis (GO:0006508)
KRC01016	1	5E-41	blast X	Masquerade-like protein precursor	*P. leniusculus*	P91777	proteolysis (GO:0006508)
KRC00950	2	2E-24	blast X	Oviductin	*A. aegypti*	Q16G08	proteolysis (GO:0006508)
KRC00371	1	6E-19	blast X	Serine protease, trypsin family	*C. psychrerythraea*	Q484F0	proteolysis (GO:0006508)
KRC00673	1	2E-26	blast X	Serine protease-like protein	*B. mori*	Q45RG0	proteolysis (GO:0006508)
KRC00890	1	1E-23	blast X	Cathepsine L-like cysteine protease	*R. prolixus*	Q8WSH3	proteolysis (GO:0006508)
KRC00933	1	1E-42	blast X	CUB-serine protease	*P. argus*	Q967X8	proteolysis (GO:0006508)
KRC00742	4	3E-18	blast X	CUB-serine protease	*P. argus*	Q967X8	proteolysis (GO:0006508)
KRC00979	1	5E-13	blast X	CUB-serine protease	*P. argus*	Q967X8	proteolysis (GO:0006508)
KRC00035	1	6E-59	blast X	Proteasome (Prosome, macropain) subunit, alpha type, 3	*X. tropicalis*	Q5PPP6	ubiquitin protein catabolic process (GO:0006511)
KRC00604	1	2E-13	blast X	Proteasome (Prosome, macropain) subunit, beta type	*X. tropicalis*	Q6P7M7	ubiquitin protein catabolic process (GO:0006511)
KRC00638	1	7E-27	blast X	Proteasome subunit beta type	*A. aegypti*	Q17IE3	ubiquitin protein catabolic process (GO:0006511)
KRC00413	1	1E-94	blast X	Proteasome subunit alpha type	*A. aegypti*	Q177R1	ubiquitin protein catabolic process (GO:0006511)
KRC01067	1	9E-71	blast X	26S protease regulatory subunit	*A. aegypti*	Q16FL0	protein catabolic process (GO:0030163)
KRC00375	3	5E-62	blast X	Anopheles stephensi ubiquitin	*A. stephensi*	Q95NR0	structural constituent of ribosome (GO:0003735)
KRC00370	1	1E-22	blast X	Methionyl aminopeptidase 2	*B. rerio*	Q7SXK1	aminopeptidase activity (GO:0004177)
KRC00009	4	6E-23	blast X	Ubiquitin/ribosomal protein S30e fusion protein	*P. dardanus*	Q6EV20	structural constituent of ribosome (GO:0003735)
KRC00878	1	7E-46	blast X	Nck-associated protein 1 (NAP 1)	*R. norvegicus*	P55161	protein binding (GO:0005515)
KRC00320	1	3E-17	blast X	Huntingtin interacting protein HYPK (Fragment)	*H. sapiens*	O75408	protein binding (GO:0005515)
KRC00343	1	2E-18	blast X	Tetratricopeptide repeat protein, putative	*A. aegypti*	Q17N59	heat shock protein binding (GO:0031072)
KRC00520	1	3E-31	blast X	Protein pellino homolog 2 (Pellino-2)	*H. sapiens*	Q9HAT8	protein binding (GO:0005515)
KRC01052	1	5E-15	blast X	14-3-3 protein sigma, gamma, zeta, beta/alpha	*A. aegypti*	Q16QZ7	protein domain specific binding (GO:0019904)
KRC00540	1	1E-29	blast X	Nuclear inhibitor of protein phosphatase type 1 (SD02428p)	*D. melanogaster*	Q9V7W9	protein binding [GO:0005515]
KRC00029	4	1E-70	blast X	Peptidyl-prolyl cis-trans isomerase A1 (Cyclophilin A1)	*R. oryzae*	P0C1H7	protein folding (GO:0006457)
KRC00030	1	2E-28	blast X	Putative cytosolic chaperonin, delta-subunit	*O. sativa*	Q9FW88	protein folding (GO:0006457)
KRC00103	1	6E-88	blast X	Chaperonin containing TCP1, subunit 3 (Gamma)	*H. sapiens*	Q59H77	protein folding (GO:0006457)
KRC00184	2	7E-59	blast X	Ppib protein (Peptidylprolyl isomerase B) (Cyclophilin B)	*B. rerio*	Q6PBW4	protein folding (GO:0006457)
KRC00185	1	1E-10	blast X	Chaperonin	*A. aegypti*	Q16U15	protein folding (GO:0006457)
KRC00523	1	9E-80	blast X	T-complex protein 1, alpha subunit(TCP-1-alpha)(CCT-alpha)	*D. antiqua*	Q4AE76	protein folding (GO:0006457)
KRC00749	1	9E-95	blast X	Chaperonin subunit 5 (Epsilon)	*R. norvegicus*	Q68FQ0	protein folding (GO:0006457)
KRC00920	1	3E-81	blast X	Hsp-90	*C. haematocheir*	Q6QR01	protein folding (GO:0006457)

7. Striated muscle contraction

Cluster ID	# EST	e-value	Program	Putative identity	Organism	Protein ID	GO Biological Process
KRC00034	1	6E-50	blast X	Hypothetical protein (Fragment)	*A. aegypti*	Q16TM8	actin cytoskeleton organization (GO:0030036)
KRC00415	3	2E-57	blast X	Alpha-2-tubulin	*G. lateralis*	O01942	microtubule-based process (GO:0007017)
KRC00781	2	1E-07	blast X	Tubulin, alpha 2	*B. rerio*	Q6TNP9	microtubule-based process (GO:0007017)
KRC00190	1	1E-18	blast X	Myosin light chain alkali	*D. simulans*	Q24654	muscle contraction (GO:0006936)
KRC00235	1	4E-23	blast X	I-connectin	*P. clarkii*	Q95YM2	muscle contraction (GO:0006936)
KRC00155	5	2E-55	blast X	Myosin heavy chain, striated muscle	*A. irradians*	P24733	striated muscle contraction (GO:0006941)
KRC00174	1	3E-19	blast X	Myosin-2 (Myosin heavy chain C) (MHC C)	*C. elegans.*	P12845	striated muscle contraction (GO:0006941)
KRC00036	3	0.000003	blast X	Troponin t, invertebrate	*A. aegypti*	Q17I95	striated muscle contraction (GO:0006941)
KRC00046	2	1E-62	blast X	Myosin heavy chain, muscle	*D. melanogaster*	P05661	striated muscle contraction (GO:0006941)
KRC00081	4	5E-22	blast X	Troponin I-b1	*D. virilis*	Q6T2X1	striated muscle contraction (GO:0006941)
KRC00122	4	3E-39	blast X	Troponin T (TnT)	*P. americana*	Q9XZ71	striated muscle contraction (GO:0006941)
KRC00055	4	2E-20	blast N	Actin mRNA, complete cds	*M. galloprovincialis*	AF157491.1	striated muscle contraction (GO:0006941)
KRC00138	1	7E-43	blast X	Troponin T	*B. mori*	Q2F5V0	striated muscle contraction (GO:0006941)
KRC00147	3	3E-73	blast X	Myosin heavy chain, nonmuscle or smooth muscle	*A. aegypti*	Q178Y3	striated muscle contraction (GO:0006941)
KRC00198	1	2E-17	blast X	Myosin heavy chain, nonmuscle or smooth muscle	*A. aegypti*	Q179E8	striated muscle contraction (GO:0006941)
KRC00216	3	1E-30	blast N	Beta-actin mRNA	*C. sapidus*	DQ084066.1	striated muscle contraction (GO:0006941)
KRC00219	5	2E-46	blast X	Slow muscle myosin S1 heavy chain (Fragment)	*H. americanus*	Q6XGZ8	striated muscle contraction (GO:0006941)
KRC00246	4	5E-36	blast N	alpha-tubulin mRNA	*X. laevis*	X07046.1	striated muscle contraction (GO:0006941)
KRC00264	1	5E-10	blast X	Coracle protein, putative	*A. aegypti*	Q170K4	striated muscle contraction (GO:0006941)
KRC00433	1	9E-47	blast N	Beta-actin (act1) mRNA	G. lateralis	L76943.1	striated muscle contraction (GO:0006941)
KRC00461	1	9E-37	blast N	Beta-actin (act2) mRNA	G. lateralis	L76530.1	striated muscle contraction (GO:0006941)
KRC00725	1	3E-24	blast N	Alpha tubulin mRNA	P. gouldii	AY855263.1	striated muscle contraction (GO:0006941)
KRC00887	1	7E-20	blast X	Slow-tonic S2 myosin heavy chain	*H. americanus*	Q6E7L5	striated muscle contraction (GO:0006941)
KRC01004	1	7E-26	blast X	Myosin heavy chain, muscle	*D. melanogaster*	P05661	striated muscle contraction (GO:0006941)
KRC01033	1	5E-13	blast X	Troponin t, invertebrate	*A. aegypti*	Q17I95	striated muscle contraction (GO:0006941)
KRC00080	2	1E-07	blast X	Slow-tonic S2 tropomyosin	*H. americanus*	Q6E7L4	striated muscle contraction (GO:0006941)
KRC00032	8	1E-32	blast X	Myosin light chain	*G. orientalis*	Q49M29	calcium ion binding (GO:0005509)
KRC00049	6	2E-36	blast X	Myosin light chain 1, putative	*A. aegypti*	Q16MS5	calcium ion binding (GO:0005509)
KRC00068	4	1E-129	blast X	Skeletal alpha1 actin (Actin, alpha 1, skeletal muscle)	*B. rerio*	Q9I8V1	ATP binding (GO:0005524)
KRC00140	7	4E-10	blast X	Sarcoplasmic calcium-binding protein 1 (SCP I)	*P. leptodactylus*	P05946	calcium ion binding (GO:0005509)
KRC00173	4	4E-13	blast X	Myosin 3 light chain	*L. obliqua*	Q5MGI7	calcium ion binding (GO:0005509)
KRC00244	1	4E-34	blast X	Sarcoplasmic calcium-binding protein 1 (SCP I)	*P. leptodactylus*	P05946	calcium ion binding (GO:0005509)
KRC00255	1	4E-35	blast X	Myosin light chain 2	*B. mori*	Q1HPS0	calcium ion binding (GO:0005509)
KRC00260	1	2E-22	blast X	Bestrophin 2,3,4	*A. aegypti*	Q17G96	calcium ion binding (GO:0005509)
KRC00304	3	2E-28	blast X	Myosin 2 light chain (Myosin 1 light chain)	*L. obliqua*	Q5MGI8	calcium ion binding (GO:0005509)
KRC00839	1	3E-16	blast X	S-layer-RTX protein	*W. recta*	Q9ZIB5	calcium ion binding (GO:0005509)

8. Signal transduction

Cluster ID	# EST	e-value	Program	Putative identity	Organism	Protein ID	GO Biological Process
KRC00367	1	4E-16	blast X	Casein kinase II subunit beta (CK II beta)	*S. frugiperda*	O76485	Wnt receptor signaling pathway (GO:0016055)
KRC00477	1	2E-98	blast X	RhoA	*T. japonicus*	Q6RWD5	small GTPase mediated signal transduction
KRC00522	1	7E-11	blast X	Ankyrin	*D. melanogaster*	Q24241	signal transduction (GO:0007165)
KRC00735	1	1E-33	blast X	Compound eye opsin BCRH1	*H. sanguineus*	Q25157	signal transduction (GO:0007165)
KRC00802	1	7E-10	blast X	Compound eye opsin BCRH1	*H. sanguineus*	Q25157	signal transduction (GO:0007165)
KRC00854	1	1E-49	blast N	G protein beta 1 subunit mRNA	*L. vannamei*	AY626793.1	intracellular signaling cascade
KRC01034	1	1E-30	blast X	Angiopoietin-related protein 1 precursor (Angiopoietin-like 1)	*M. musculus*	Q640P2	signal transduction (GO:0007165)
KRC00023	2	2E-24	blast X	Serum lectin isoform 2	*S. salar*	Q800Z5	cell surface receptor linked signal transduction

9. Structural constituent of cuticle

Cluster ID	# EST	e-value	Program	Putative identity	Organism	Protein ID	GO Biological Process
KRC00014	1	2E-08	blast X	Crustin-like peptide (Fragment)	*P. japonicus*	Q75WB3	structural constituent of cuticle (GO:0042302)
KRC00308	4	3E-18	blast X	Arthrodial cuticle protein AMP13.4	*C. sapidus*	Q2V6U3	structural constituent of cuticle (GO:0042302)
KRC00330	1	1E-22	blast X	Cuticle protein AMP1A (HA-AMP1A)	*H. americanus*	P81384	structural constituent of cuticle (GO:0042302)
KRC00432	1	3E-16	blast X	Calcified cuticle protein CP14.1	*C. sapidus*	Q2V6T9	structural constituent of cuticle (GO:0042302)
KRC00536	1	1E-15	blast X	Exoskeletal protein HACP188 (Fragment)	*H. americanus*	Q7M497	structural constituent of cuticle (GO:0042302)
KRC00566	5	1E-21	blast X	Arthrodial cuticle protein AMP16.3	*C. sapidus*	Q2PPJ9	structural constituent of cuticle (GO:0042302)
KRC00584	1	6E-11	blast X	Cuticular protein isoform HACP4.6b	*H. americanus*	Q7M4A1	structural constituent of cuticle (GO:0042302)
KRC00620	2	4E-16	blast X	Cuticular protein (Fragment)	*T. tridentatus*	Q3V6R9	chitin binding (GO:0008061)
KRC00651	3	1E-16	blast X	Cuticle protein AM1159 (CPAM1159)	*C. pagurus*	P81576	structural constituent of cuticle (GO:0042302)
KRC00720	1	7E-21	blast X	Cuticle protein AMP4 (HA-AMP4)	*H. americanus*	P81388	structural constituent of cuticle (GO:0042302)
KRC00902	2	3E-13	blast X	Calcification-associated peptide-1 precursor	*P. clarkii*	Q7YUE2	structural constituent of cuticle (GO:0042302)
KRC00945	1	3E-13	blast X	Cuticle protein AMP5 (HA-AMP5)	*H. americanus*	P81389	structural constituent of cuticle (GO:0042302)
KRC00996	1	2E-16	blast X	Cuticle protein, putative	*A. aegypti*	Q16XV0	structural constituent of cuticle (GO:0042302)
KRC01076	2	3E-17	blast X	BCS-1	*B. amphitrite*	Q9NDT7	structural constituent of cuticle (GO:0042302)

10. Cell growth, proliferation and adhesion

Cluster ID	# EST	e-value	Program	Putative identity	Organism	Protein ID	GO Biological Process
KRC00152	1	0.000007	blast X	Intercellular adhesion molecule 2 variant (Fragment)	*H. sapiens*	Q59ED3	cell-cell adhesion (GO:0016337)
KRC00410	1	6E-18	blast X	Rapsynoid (Fragment)	*A. aegypti*	Q16H64	asymmetric cell division
KRC00527	2	4E-47	blast X	Defender against programmed cell death	*A. gambiae*	Q6TEC7	cell proliferation (GO:0008283)
KRC00680	3	2E-22	blast X	Nucleolar protein NOP5 (Nucleolar protein 5)	*H. sapiens*	Q9Y2X3	cell growth (GO:0016049)
KRC00872	1	1E-17	blast X	Nuclear autoantigenic sperm protein (Histone-binding)	*R. norvegicus*	Q66HD3	cell proliferation (GO:0008283)
KRC00925	1	5E-38	blast X	Cell death-regulatory protein GRIM19	*B. mori*	Q2F619	cell proliferation (GO:0008283)
KRC01026	1	1E-25	blast X	Headcase protein homolog (hHDC)	*H. sapiens*	Q9UBI9	regulation of progression cell cycle (GO:0000074)
KRC01057	1	8E-08	blast X	Cysteine-rich motor neuron 1 protein precursor (CRIM-1)	*M. musculus*	Q9JLL0	regulation of cell growth (GO:0001558)

11. Ion Binding

Cluster ID	# EST	e-value	Program	Putative identity	Organism	Protein ID	GO Biological Process
KRC00612	1	7E-24	blast X	Salivary selenoprotein M	*I. scapularis*	Q4PMN9	selenium binding (GO:0008430)
KRC00669	1	2E-36	blast X	Selenoprotein (Fragment)	*A. gambiae*	Q8WR40	selenium binding (GO:0008430)
KRC00505	1	5E-28	blast X	Cysteine-rich intestinal protein	*H. medicinalis*	Q8I8C7	zinc ion binding (GO:0008270)
KRC00773	1	4E-18	blast X	Zinc-binding alcohol dehydrogenase-like protein	*O. dioica*	Q66S07	zinc ion binding (GO:0008270)
KRC00777	1	2E-23	blast X	Nuclear protein UKp68	*R. norvegicus*	Q7TSK6	zinc ion binding (GO:0008270)

12. Hypotetical protein

Cluster ID	# EST	e-value	Program	Putative identity	Organism	Protein ID	GO Biological Process
KRC00006	1	2E-46	blast X	Uncharacterized conserved secreted protein	*I. loihiensis*	Q5QUQ1	
KRC00097	2	8E-13	blast X	Hypothetical protein	*C. sonorensis*	Q66U68	
KRC00104	1	3E-30	blast X	Hypothetical protein	*X. tropicalis*	Q6P645	
KRC00242	1	3E-63	blast X	Hypothetical protein	*G. gallus*	Q5ZJ82	
KRC00251	5	3E-50	blast X	Hypothetical protein (Fragment)	*B. rerio*	Q0P429	
KRC00321	1	3E-11	blast X	Coiled-coil domain-containing protein 55	*B. taurus*	Q2KIC0	
KRC00331	1	1E-20	blast X	Hypothetical protein	*X. laevis*	Q3B8C3	
KRC00509	1	3E-22	blast X	Hypothetical protein	*A. aegypti*	Q17EJ4	
KRC00635	2	1E-30	blast X	Hypothetical protein (Fragment)	*C. albicans*	Q59JR6	
KRC00746	1	3E-11	blast X	Transmembrane and coiled-coil domains 4	*M. musculus*	Q91WU4	

13. Mitochondrial genes

Cluster ID	# EST	e-value	Program	Putative identity	Organism	Protein ID	GO Biological Process
KRC00003	88	0	blastN	16S ribosomal RNA gene	*E. superba*	M98483.1	
KRC00399	5	1E-81	blastX	ATP synthase subunit 6 (ATPase6)	*E. superba*	Q76L76	
KRC00062	33	0	blastN	cytochrome c oxidase subunit I (COI) gene	*E. superba*	AF177182.1	
KRC00782	1	3E-78	blastX	Cytochrome oxidase subunit I (COI)	*E. superba*	Q76L79	
KRC00001	20	1E-105	blastX	Cytochrome oxidase subunit II (COII)	*E. superba*	Q76L78	
KRC00359	6	1E-97	blastX	Cytochrome oxidase subunit III (COIII)	*E. superba*	Q76L75	
KRC00175	6	1E-104	blastX	Cytochrome b apoenzyme (cytb)	*E. superba*	Q76L69	
KRC00182	3	1E-135	blastN	NADH dehydrogenase subunit 1 (ND1)	*E. superba*	AF281263.1	
KRC00366	4	3E-49	blastX	NADH dehydrogenase subunit 2 (ND2)	*E. superba*	Q76L80	
KRC00808	1	4E-20	blastX	Mitochondrial NADH-ubiquinone oxidoreductase AGGG subunit	*A. aegypti*	Q1HR97	
KRC00591	4	8E-40	blastX	NADH dehydrogenase subunit 3 (ND3)	*E. superba*	Q76L74	
KRC00940	1	2E-81	blastN	mitochondrial DNA, trnM to rrnS gene region	*E. superba*	AB084378.1	
KRC00391	1	1E-135	blastN	mitochondrial DNA, trnM to rrnS gene region	*E. superba*	AB084378.1	
KRC00677	1	1E-107	blastN	mitochondrial DNA, trnM to rrnS gene region	*E. superba*	AB084378.1	

14. RNA 18S e 28S contaminants

Cluster ID	# EST	e-value	Program	Putative identity	Organism	Protein ID	GO Biological Process
KRC00005	1	1E-158	blastn	28S large subunit ribosomal RNA gene	*E. eximia*	DQ079787.1	ribosomal RNA gene
KRC00234	1	1E-148	blastn	18S ribosomal RNA gene	*E. superba*	DQ201509.1	ribosomal RNA gene
KRC00241	3	1E-172	blastn	28S large subunit ribosomal RNA gene	*E. superba*	AF169700.1	ribosomal RNA gene
KRC00040	2	1E-148	blastn	18S ribosomal RNA gene	*E. superba*	DQ201509.1	ribosomal RNA gene
KRC00844	2	1E-151	blastn	18S ribosomal RNA gene	*E. superba*	DQ201509.1	ribosomal RNA gene
KRC00324	1	2E-93	blastn	18S ribosomal RNA gene	*E. superba*	DQ201509.1	ribosomal RNA gene
KRC00877	1	1E-128	blastn	Large subunit ribosomal RNA gene	*E. superba*	AF169717.2	ribosomal RNA gene
KRC00291	1	1E-40	blastn	Large subunit ribosomal RNA gene	*E. superba*	AF169717.2	ribosomal RNA gene
KRC00070	1	8E-68	blastn	Large subunit ribosomal RNA gene	*E. superba*	AF169717.2	ribosomal RNA gene
KRC00041	2	4E-22	blastn	18S ribosomal RNA gene	*P. fluviatilis*	AF518195.1	ribosomal RNA gene
KRC00067	3	1E-62	blastn	28S ribosomal RNA gene	*S. empusa*	AY210842.1	ribosomal RNA gene
KRC00333	3	0	blastn	Small subunit ribosomal RNA gene	*E. superba*	AY672801.1	ribosomal RNA gene
KRC00188	5	0	blastn	Small subunit ribosomal RNA gene	*E. superba*	AY672801.1	ribosomal RNA gene
KRC00526	3	1E-136	blastn	Small subunit ribosomal RNA gene	*E. superba*	AY672801.1	ribosomal RNA gene
