# Supplementary material for: Systematic sequencing of mRNA from the Antarctic krill (Euphausia superba) and first tissue specific transcriptional signature
Source: BMC Genomics. 2008 Jan 28;9:45. doi: 10.1186/1471-2164-9-45 (PMC2270838; doi:10.1186/1471-2164-9-45)
Supplement: Additional file 2 — Quantitative RT-PCR validation. Forward and reverse primer pairs for ten tested transcripts are detailed at the top. Relative expression values resulting from the quantitative real-time PCR are in the middle and related data plots are also reported. For each transcriptional profiling we have associated the EST counting obtained from the cDNA libraries systematic sequencing. [file 1471-2164-9-45-S2.PDF]

Additional File 2

Forward and reverse primers used for qRT-PCR

# Consensus	Description	Forward Primer (5'-3')	Reverse Primer (5'-3')
KRC00735	Compound eye opsin BCRH1	TGACAGTCCACATGCCATGGT	TCTGCGAAGGCTCCTATGCC
KRC00032	Myosin light chain	GTATCATCGGCATGCTCTGCG	ACTGGAACCCTCATTGCCCG
KRC00155	Myosin heavy chain	TGAGGCTGAACTGGCCAAACT	GACATCTCGCCAACAGCATCA
KRC00566	Arthrodial cuticle protein AMP16.3	CAGTCTCCATTGGTCCCCGT	ACCCCCTGTGCAGCGAATT
KRC00319	Tail muscle elongation factor 1 gamma	GGTGTCTTCGGTGAGGATGGT	GGTGCGTCAGCATCAAGCTT
KRC00338	Cellular retinoic acid/retinol binding protein	CGCATACGTCCCAGTACTTCC	CATCGAAGCCCTCAGAGCTG
KRC00786	Eukaryotic initiation factor 4A	TCCAACAGGTTTCCCTTGTCA	TGGCAACACCTTTCCTACCAA
KRC00208	Transport protein SEC61 subunit gamma	TGCACCAAGCCAGACAGAAAG	TCCTAGGCCCCCACAATGA
KRC00402	Chromodomain helicase DNA binding protein	CCGCTGCCTCCATTATTAGCC	TGCTTCTTGAGGTGGCATTCC
KRC00160	Voltage-dependent calcium channel	GCGGATGTTGGTTTCATCCC	CCAGGTCAAGCCCAGAGGAA

Relative Expression values (RQ)

# Consensus	Description	Tissues	RQ	RQ min	RQ max
KRC00735	Compound eye opsin BCRH1	Head	17297.06	15866.30	18856.85
		Abdomen	1.00	0.36	2.76
		Photophores	86.42	66.51	112.27
		Thoracopods	3.06	1.93	4.86
KRC00032	Myosin light chain	Head	0.12	0.11	0.15
		Abdomen	1.00	0.89	1.13
		Photophores	0.17	0.15	0.20
		Thoracopods	0.15	0.13	0.16
KRC00155	Myosin heavy chain	Head	0.43	0.39	0.46
		Abdomen	1.00	0.88	1.13
		Photophores	0.40	0.35	0.46
		Thoracopods	2.30	1.76	3.01
KRC00566	Arthrodial cuticle protein AMP16.3	Head	3500.00	3166.70	3868.38
		Abdomen	1.00	0.86	1.16
		Photophores	3.03	1.78	5.15
		Thoracopods	1.85	1.22	2.79
KRC00319	Tail muscle elongation factor 1 gamma	Head	2.16	1.86	2.51
		Abdomen	1.00	0.81	1.23
		Photophores	2.29	2.03	2.60
		Thoracopods	0.87	0.77	0.99
KRC00338	Cellular retinoic acid/retinol binding protein	Head	0.90	0.83	0.96
		Abdomen	1.00	0.91	1.09
		Photophores	6.04	5.06	7.21
		Thoracopods	2.02	1.56	2.37
KRC00786	Eukaryotic initiation factor 4A	Head	1.40	1.23	1.61
		Abdomen	1.00	0.87	1.15
		Photophores	2.08	1.79	2.40
		Thoracopods	1.65	1.35	2.03
KRC00208	Transport protein SEC61 subunit gamma	Head	6.06	4.73	7.77
		Abdomen	1.00	0.72	1.39
		Photophores	0.07	0.04	0.10
		Thoracopods	0.84	0.75	0.94
KRC00402	Chromodomain helicase DNA binding protein	Head	2.58	2.23	2.99
		Abdomen	1.00	0.71	1.42
		Photophores	3.28	2.84	3.77
		Thoracopods	2.89	2.38	3.50
KRC00160	Voltage-dependent calcium channel	Head	0.29	0.23	0.38
		Abdomen	1.00	0.57	1.35
		Photophores	0.21	0.18	0.25
		Thoracopods	0.18	0.14	0.23

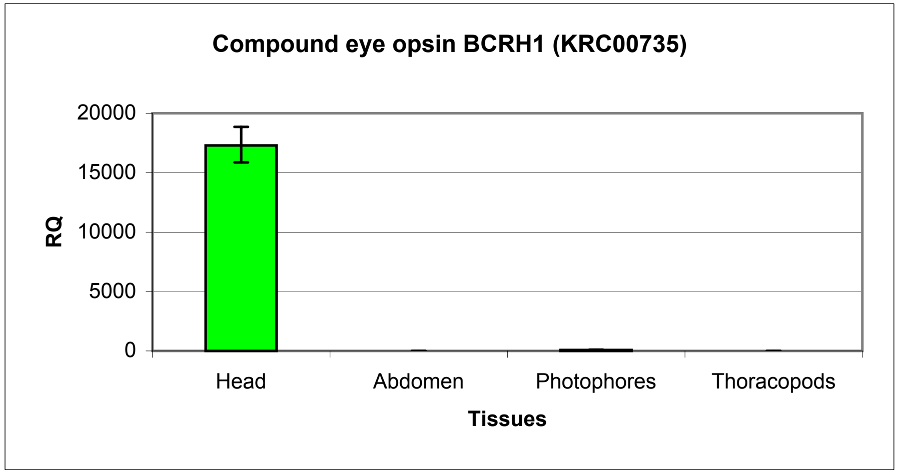

	Head	Abdomen	Photophores	Thoracopods
**KRC00735**	1	0	0	0

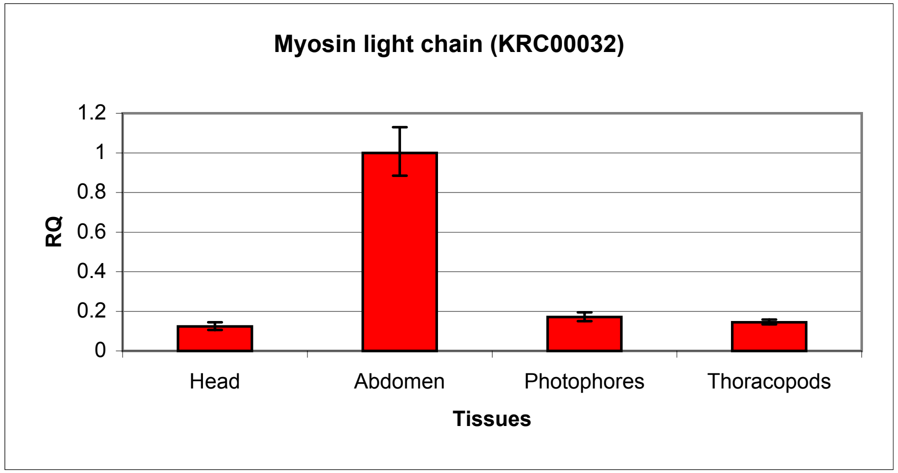

	Head	Abdomen	Photophores	Thoracopods
**KRC00032**	0	8	0	0

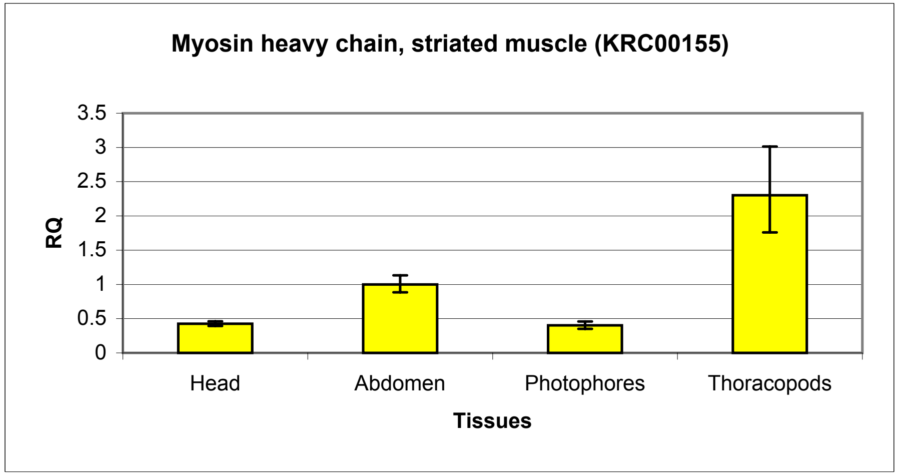

	Head	Abdomen	Photophores	Thoracopods
**KRC00155**	0	3	0	2

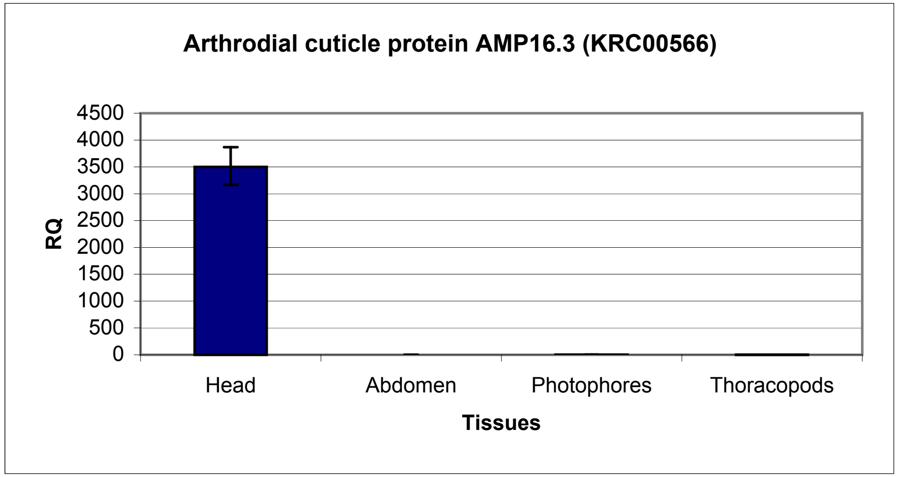

	Head	Abdomen	Photophores	Thoracopods
**KRC00566**	5	0	0	0

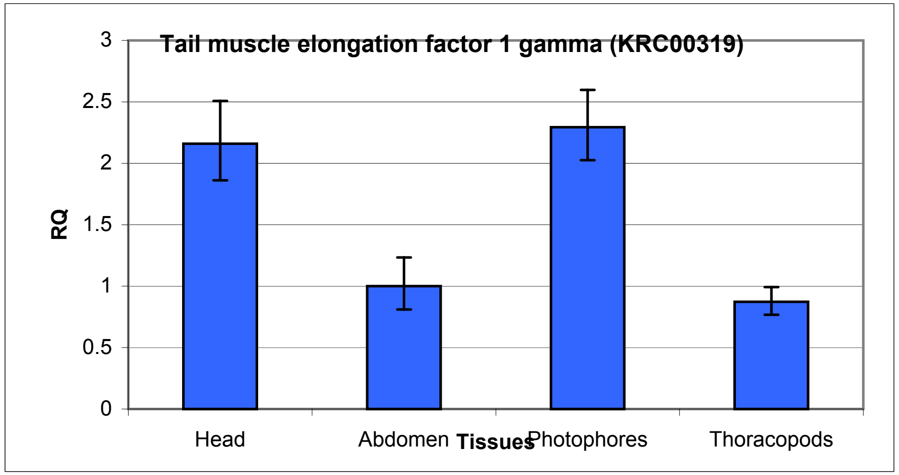

	Head	Abdomen	Photophores	Thoracopods
KRC00319	0	0	2	0

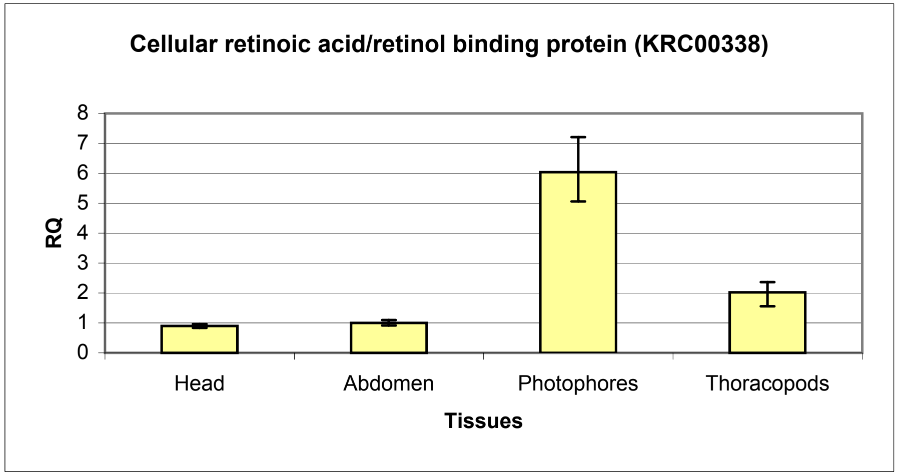

	Head	Abdomen	Photophores	Thoracopods
KRC00338	0	0	1	0

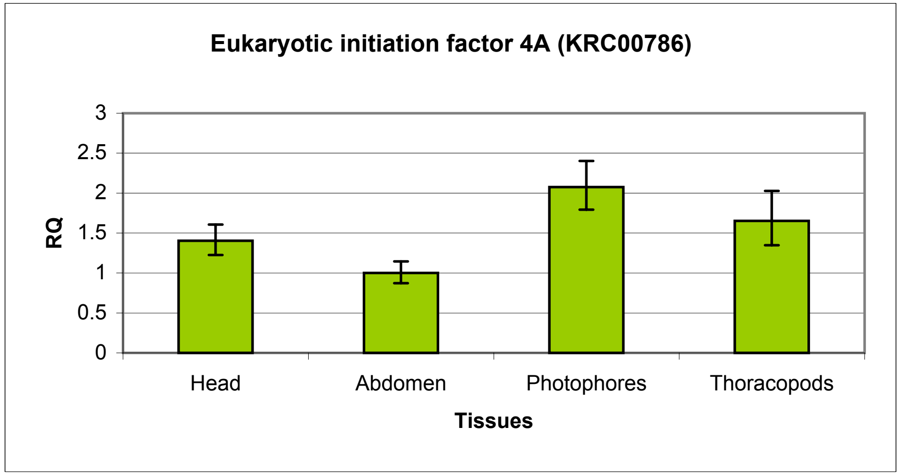

	Head	Abdomen	Photophores	Thoracopods
KRC00786	1	0	0	1

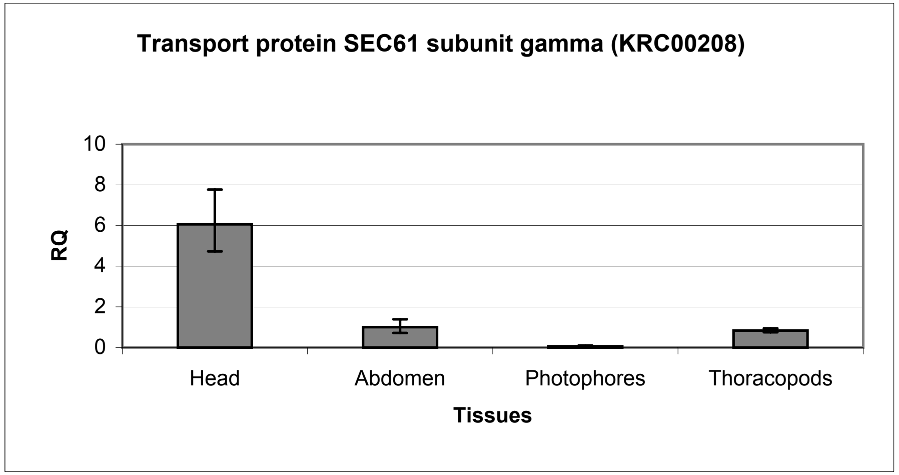

	Head	Abdomen	Photophores	Thoracopods
KRC00208	1	0	0	1

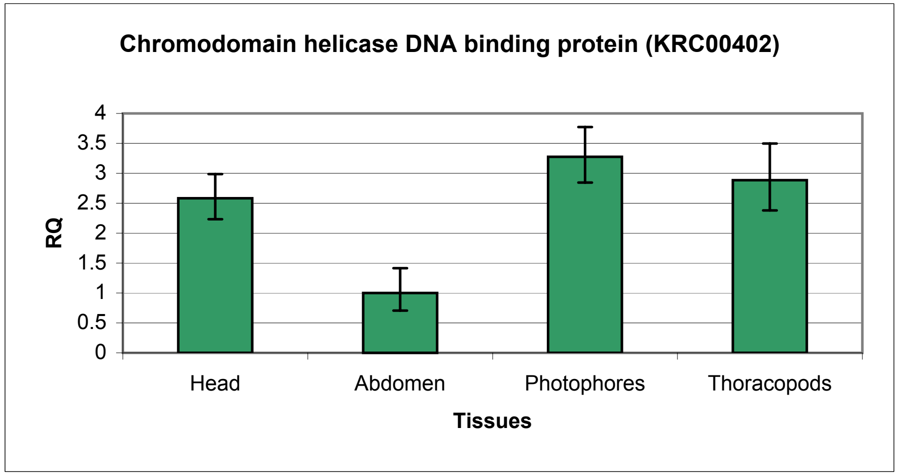

	Head	Abdomen	Photophores	Thoracopods
**KRC00402**	0	0	1	0

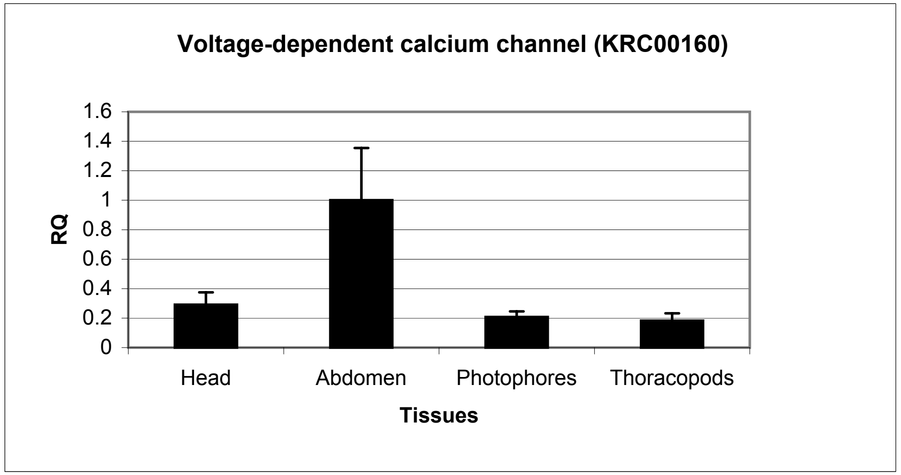

	Head	Abdomen	Photophores	Thoracopods
**KRC00160**	0	1	0	0
